# Ophiostomatoid fungi associated with *Ips subelongatus*, including eight new species from northeastern China

**DOI:** 10.1186/s43008-019-0025-3

**Published:** 2020-01-31

**Authors:** Zheng Wang, Ya Liu, Huimin Wang, Xianjing Meng, Xuewei Liu, Cony Decock, Xingyao Zhang, Quan Lu

**Affiliations:** 1 Key Laboratory of Forest Protection, National Forestry and Grassland Administration; Research Institute of Forest Ecology, Environment and Protection, Chinese Academy of Forestry, Beijing, 100091 China; 2 Wuqing Forestry Bureau, Tianjin, 301700 China; 3 Mycothèque de l’Université Catholique de Louvain (MUCL), Earth and Life Institute, Microbiology, B-1348 Louvain-la-Neuve, Belgium

**Keywords:** *Ceratocystiopsis*, *Endoconidiophora*, *Leptographium*, *Ophiostoma*, Taxonomy

## Abstract

*Ips subelongatus* is a major pest that infects larch plantations over large areas of northern and northeastern China. *Ips* species are closely associated with ophiostomatoid fungi that are morphologically well-adapted for dispersal by beetles. These associations result in important threat for coniferous forests worldwide. The aim of this study was to characterize the ophiostomatoid communities associated with *I. subelongatus* infesting *Larix* species and sympatric *Pinus sylvestris* var. *mongolica* in northeastern China forests. Morphological and multilocus phylogenetic approaches (based on six markers: ITS, LSU, 60S, β-tubulin, EF-1α, and CAL gene regions) allowed identifying 14 species of four genera (*Ceratocystiopsis*, *Endoconidiophora*, *Leptographium* and *Ophiostoma*). Eight species are showed to be new to science. Most strains resided in two *Ophiostoma* species complexes, viz. the *O. clavatum* and the *O. ips* complexes, all together accounting for 76.8% of all isolates. *Ophiostoma hongxingense* sp. nov., *O. peniculi* sp. nov., and *O. subelongati* sp. nov. (*O. clavatum* complex) and *O. pseudobicolor* sp. nov. (*O. ips* complex) were the four dominant species. The ophiostomatoid communities associated with larch bark beetles, *I. cembrae* and *I. subelongatus*, in Europe and Asia, China and Japan, also were compared. These comparisons showed distinct, specific assemblage patterns.

## INTRODUCTION

Globalization has hastened the emergence of tree pests, prompting the urgent need for a global strategy to manage the vitally important issues of forest pests (Wingfield et al. 2015). Bark beetles (*Curculionidae: Scolytinae*) are phloem-boring insects, some of which are the primary pests responsible for considerable mortality of coniferous forests in the northern hemisphere (Raffa et al. 2015). In Eurasia, species of the bark beetle genera *Ips*, *Tomicus*, and *Dendroctonus* have received a great deal of attention because of the damage they cause to local forest ecosystems and/or to tree plantations (Miao et al. 2001, Grégoire & Evans 2004, Vega & Hofstetter 2014).

*Ips subelongatus* is a widely distributed bark beetle species in east Asia, spanning over Japan, Korea, Northern China, Mongolia, and the Russian Far East. It infests numerous *Larix* species (*Pinaceae*) including *L. gmelinii*, *L. olgensis*, *L. principis-rupprechtii*, *L. kaempferi*, *L. sibirica*, and sometimes *Pinus* spp. In China, *I. subelongatus* mainly infects three allopatric larches (Yang et al. 2007); they are *L. gmelinii* in the Da Xing’an and Xiao Xing’an mountain ranges in the Inner Mongolia Autonomous Region and Heilongjiang Province, *L. olgensis* in southeastern Heilongjiang Province, the Chang Bai mountain range in Jilin and Liaoning Provinces, and *L. principis-rupprechtii* in middle Inner Mongolia as well as Beijing, Hebei, and Shanxi Provinces. These larches constitute the main component of one of the largest forested area in northeastern China.

The Asian eight spined larch bark beetle has commonly been considered a secondary pest that mainly attacks dying trees or colonizes stock logs (Stauffer et al. 2001, Yamaoka et al. 1998). However, extensive infestations of larches with high insect density, high tree mortality rates, and subsequent forest decline have been noticed in these areas since the 1980s (Yin et al. 1984, Yu 1992, Zhang et al. 2007). Because of possible incidental introductions through the timber trade, *I. subelongatus* was presented in the EPPO alert list A2 as an important pest that is affecting coniferous trees in native regions and which represents a threat to non-native regions (EPPO 2005).

The association between beetles and fungi commonly plays an important role in the success of beetle colonization (Kirisits 2004). One of the most important beetle associated fungal groups are the ophiostomatoid fungi (Wingfield et al. 1993, Kirisits 2004). Ophiostomatoid fungi are an assemblage of species that share similar morphological and ecological traits. They belong mainly to the order *Ophiostomatales* (*Sordariomycetidae*, *Sordariomycetes*, *Ascomycota*), which includes the genera *Ophiostoma*, *Leptographium*, *Sporothrix*, *Raffaelea*, and *Ceratocystiopsis*, and to the order *Microascales* (*Hypocreomycetidae*, *Sordariomycetes*, *Ascomycota*), which includes *Ceratocystis*, *Graphium* and *Endoconidiophora* (De Beer & Wingfield 2013, De Beer et al. 2014, De Beer et al. 2016). These fungi are assumed to be closely associated with bark beetles because of their morphological and ecological characteristics (Kirisits 2004). Some of these fungi are known as trees pathogens [e.g. *Ophiostoma ulmi* and *O. novo-ulmi* causing the Dutch elm disease (De Hoog et al. 1974, Brasier 1991), *Leptographium wageneri*, responsible for the black root disease (Harrington & Cobb 1988), or *Endoconidiophora fujiensis*, which could kill mature larch trees (Yamaoka et al. 1998)], but the majority are blue stain agents of timber, causing economic and ecological losses to the forestry industry.

The ophiostomatoid fungi associated with *I. subelongatus* in Japan have been investigated systematically by Aoshima (1965), Westhuizen et al. (1995), Yamaoka et al. (1998, Yamaoka et al. 2009), Chung et al. (2006), Masuya et al. (2009) and Ando et al. (2016). Yamaoka (2017) has summarized the taxonomic knowledge of these fungi in Japan, where at least 12 species were reported to be associated with *I. subelongatus*, including five species of *Ophiostoma*, three of *Grosmannia*, two of *Endoconidiophora*, and one of *Ceratocystiopsis* and *Graphilbum* (Additional file 1: Table S1).

The association of ophiostomatoid fungi with *I. subelongatus* has been reported sporadically in China. So far, ten species have been recorded in northeastern China, viz. *Endoconidiophora fujiensis*, *Graphium laricis*, *Gr. carbonarium*, *Leptographium altius*, *L. innermongolicum*, *L. manifestum*, *L. taigense*, *L. zhangii*, *Ophiostoma piceae* and *O. olgensis* (Paciura et al. 2010a, b; Meng et al. 2015; Liu et al. 2016, 2017; Wang et al. 2016; see Additional file 1: Table S1). However, a comprehensive study of their diversity, distribution and host ranges, and ecological role is still lacking.

The primary goal of the present study is to fill this gap in the knowledge of the communities of ophiostomatoid fungi on larch species in northeastern China, based on an extensive field survey and using integrated morphological observations and multilocus DNA sequence data to characterize the species. The occurrences of the ophiostomatoid communities in larch forests are analyzed. In addition to species diversity, communities among European and Asian eight spined larch bark beetles are also compared.

## MATERIALS AND METHODS

### Collection of samples and isolation of fungi

**Fig. 1:**
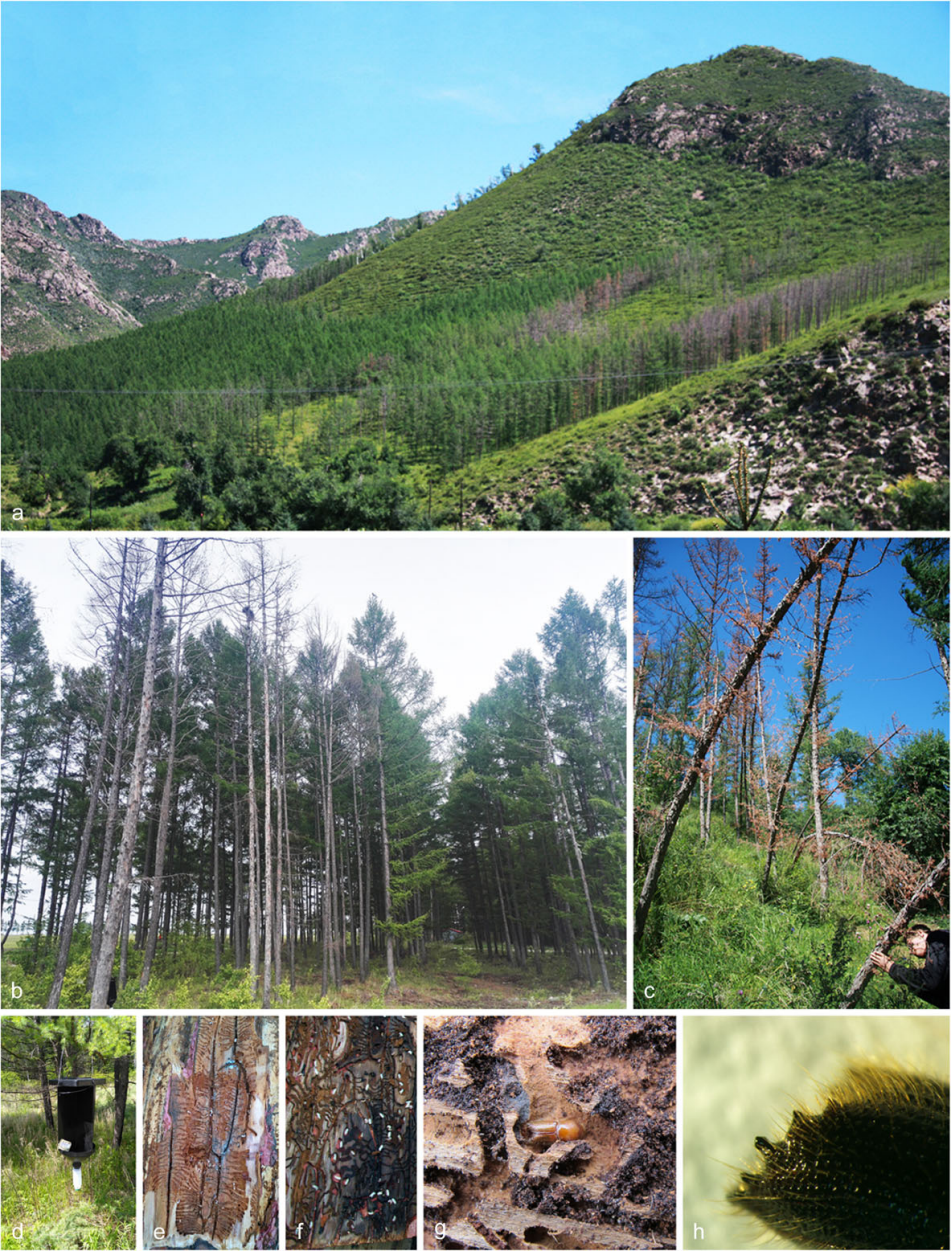
**a–c**. Disease symptoms on larch infested by *Ips subelongatus* and ophiostomatoid fungi in northeastern China; **d.** Cross barrier type traps used in fields for monitoring the occurrence and population of *I. subelongatus*; **e–g.** Larvae, pupae, and adult of *I. subelongatus* in galleries on larch; **h**. Spines of *I. subelongatus*

Fungi were isolated from adults of *Ips subelongatus* and their breeding galleries in *Larix gmelinii, L. olgensis, L. principis-rupprechtii* and, in some cases, in *Pinus sylvestris* var. *mongolica* during the beetle’s second mass flight period, which is from July to August, at 20 locations in northeastern China, including the three provinces of Heilongjiang, Liaoning, and Jilin and the autonomous region of Inner Mongolia (Fig. 1), from year 2010 to 2017. At each sampling location, beetle infested bark areas were collected from three to five dying trees or stock logs. Adults of *I. subelongatus* and their galleries were placed individually in sterile Eppendorf tubes and envelope bags, respectively. These organisms were subsequently stored at 4 °C until fungal isolation. Ten later-developed galleries in phloem and 10 adult beetles collected from these galleries at each location were used for fungal isolation. Galleries were disinfected for 1 min with 1.5% sodium hypochlorite, rinsed with sterile water three times, then cut into tissue pieces approximately 3 × 3 mm^2^ in a laminar flow hood, and five pieces of each gallery were selected and transferred onto 2% malt extract agar (MEA, malt extract and agar: AoBoXing Company Ltd., Beijing, China; recipe: add 20 g malt extract and 20 g agar per 1000 mL water). Adult beetles were crushed on the surface of 2% MEA without superficial disinfection. After a period of incubation at 25 °C in dark, all strains were purified by single-spore isolations and/or mycelium apex and routinely grown on 2% MEA. After an initial analysis of macro- and microscopical characteristics, representative strains of each morphotype were selected for further in-depth morphological, physiological, and molecular studies. All strains were deposited in the culture collection of the Chinese Academy of Forestry (CXY) (Table 1). Representatives were also deposited at the China Forestry Culture Collection Centre (CFCC) and the Mycothèque of the Université Catholique de Louvain, Belgium (BCCM/MUCL) (Table 1).

**Table 1:** Strains of ophiostomatoid fungi sequenced and used for morphological and phylogenetic analysis in this study

Taxon	Species^1^	Strain no.^2.3.4^	Host	Locality	GenBank number^5^			
					ITS/LSU/60S	βT	EF-1α	CAL
1	***Ophiostoma genhense***	CFCC 52675 (CXY 2001) T	*Larix gmelinii*	Genhe, Inner Mongolia	MK748199	MN896026	MN896074	MN896102
		CFCC 52676 (CXY 2002)	*L. gmelinii*	Genhe, Inner Mongolia	N/A	MN896028	MN896073	MN896101
2	***O. hongxingense***	CFCC 52695 (CXY 2021) T	*L. gmelinii*	Harbin, Heilongjiang	MK748194	MN896027	MN896068	MN896089
		CFCC 52696 (CXY 2022)	*L. gmelinii*	Harbin, Heilongjiang	N/A	MN896030	MN896067	MN896090
		CXY 1905	*L. gmelinii*	Harbin, Heilongjiang	N/A	MN896029	MN896066	MN896087
		CXY 1906	*L. gmelinii*	Harbin, Heilongjiang	N/A	MN896036	MN896070	MN896088
		CXY 1907	*L. gmelinii*	Harbin, Heilongjiang	N/A	MN896037	MN896069	MN896091
3	***O. lotiforme***	CFCC 52691 (CXY 2017 = MUCL 55165) T	*Pinus sylvestris* var. *mongolica*	Hailar, Inner Mongolia	MK748185	MN896044	N/A	N/A
		CFCC 52692 (CXY 2018)	*P. sylvestris* var. *mongolica*	Hailar, Inner Mongolia	MK748201	MN896045	N/A	N/A
4	*O. minus*	CFCC 52697 (CXY 2023)	*L. gmelinii*	Arongqi, Inner Mongolia	MK748202	MN896050	N/A	N/A
		CFCC 52698 (CXY 2024 = MUCL 55157)	*P. sylvestris* var. *mongolica*	Hailar, Inner Mongolia	MK748187	MN896051	N/A	N/A
5	***O. multisynnematum***	CFCC 52677 (CXY 2003) T	*L. gmelinii*	Genhe, Inner Mongolia	MK748196	MN896048	MN896071	MN896103
		CFCC 52678 (CXY 2004)	*L. gmelinii*	Genhe, Inner Mongolia	N/A	MN896049	MN896072	MN896104
6	*O. olgensis*	CFCC 52699 (CXY 2025)	*L. gmelinii*	Genhe, Inner Mongolia	MK748204	MN896046	N/A	N/A
		CFCC 52700 (CXY 2026)	*L. gmelinii*	Genhe, Inner Mongolia	MK748195	MN896047	N/A	N/A
		CXY 1908	*L. gmelinii*	Harbin, Heilongjiang	MK748203	MN896034	N/A	N/A
		CXY 1909	*L. gmelinii*	Yichun, Heilongjiang	MK748205	MN896033	N/A	N/A
7	***O. peniculi***	CFCC 52687 (CXY 2013) T	*L. gmelinii*	Genhe, Inner Mongolia	MK748198	MN896035	MN896063	MN896086
		CFCC 52688 (CXY 2014)	*L. gmelinii*	Genhe, Inner Mongolia	N/A	MN896038	MN896061	MN896084
		CXY 1904	*L. gmelinii*	Genhe, Inner Mongolia	N/A	MN896040	MN896062	MN896085
8	***O. pseudobicolor***	CFCC 52683 (CXY 2009) T	*L. gmelinii*	Genhe, Inner Mongolia	MK748188	MN896043	N/A	N/A
		CFCC 52684 (CXY 2010)	*L. gmelinii*	Genhe, Inner Mongolia	MK748190	MN896041	N/A	N/A
		CFCC 52685 (CXY 2011 = MUCL 55168)	*L. principis-rupprechtii*	Chifeng, Inner Mongolia	MK748191	MN896039	N/A	N/A
		CFCC 52686 (CXY 2012 = MUCL 55174)	*L. gmelinii*	Mohe, Heilongjiang	MK748193	MN896042	N/A	N/A
		CXY 1910	*L. principis-rupprechtii*	Chifeng, Inner Mongolia	MK748192	MN896058	N/A	N/A
		CXY 1911 (MUCL 55170)	*L. gmelinii*	Tahe, Heilongjiang	MK748189	MN896057	N/A	N/A
9	*O. rufum*	CFCC 52681 (CXY 2007)	*L. gmelinii*	Genhe, Inner Mongolia	MK748197	MN896031	MN896075	MN896095
		CFCC 52682 (CXY 2008)	*L. gmelinii*	Genhe, Inner Mongolia	N/A	MN896032	MN896076	MN896098
10	***O. subelongati***	CFCC 52693 (CXY 2019) T	*L. gmelinii*	Harbin, Heilongjiang	MK748200	MN896055	MN896064	MN896092
		CFCC 52694 (CXY 2020)	*L. gmelinii*	Harbin, Heilongjiang	N/A	MN896054	MN896065	MN896093
11	***O. xinganense***	CFCC 52679 (CXY 2005) T	*L. gmelinii*	Genhe, Inner Mongolia	MK748186	MN896056	MN896078	MN896097
		CFCC 52680 (CXY 2006)	*L. gmelinii*	Genhe, Inner Mongolia	N/A	MN896059	MN896079	MN896094
		CXY 1901	*L. gmelinii*	Genhe, Inner Mongolia	N/A	MN896060	MN896077	MN896096
		CXY 1902	*L. gmelinii*	Genhe, Inner Mongolia	N/A	MN896026	MN896080	MN896099
		CXY 1903	*L. gmelinii*	Genhe, Inner Mongolia	N/A	MN896028	MN896081	MN896100
12	*Ceratocystiopsis* cf. *pallidobrunnea*	CFCC 52689 (CXY 2015)	*P. sylvestris* var. *mongolica*	Hailar, Inner Mongolia	MN892641	N/A	N/A	N/A
		CFCC 52690 (CXY 2016)	*P. sylvestris* var. *mongolica*	Hailar, Inner Mongolia	MN892642	N/A	N/A	N/A
13	*Leptographium zhangii*	CFCC 52701 (CXY 2027)	*L. gmelinii*	Genhe, Inner Mongolia	N/A	N/A	MN896082	N/A
		CFCC 52702 (CXY 2028)	*L. gmelinii*	Genhe, Inner Mongolia	N/A	N/A	MN896083	N/A
14	*Endoconidiophora fujiensis*	CXY 1912	*L. gmelinii*	Yichun, Heilongjiang	MN896105	N/A	N/A	N/A
		CXY 1913	*L. gmelinii*	Yichun, Heilongjiang	MN896106	N/A	N/A	N/A

1. Species named in black bold are novel species in this study

2. *CFCC* China Forestry Culture Collection Center, Beijing, China

3. *CXY* the culture collection of the Chinese Academy of Forestry

4. T = ex-holotype isolates

5. *ITS* Internal transcribed spacer regions 1 and 2 of the nuclear ribosomal DNA operon, including the 5.8S region, *LSU* Large subunit of the nrDNA, *60S* partial 60S ribosomal protein RPL10 gene, *βT* the β-tubulin gene region, *EF-1α* the transcription elongation factor-1α gene region, *CAL* the calmodulin gene region

### Morphological and cultural studies

Morphological structures were observed and recorded using an Olympus BX51 microscope, Olympus SZX16 stereomicroscope, and Olympus DP70 digital camera (Olympus, Centre Valley, PA, USA). For the strains selected as holotypes, the lengths and widths of 30 reproductive structures per strain were measured. The average (mean), standard deviation (SD), minimum (min), and maximum (max) measurements are presented as the (min–) (mean − SD)–(mean + SD) (−max).

For growth rate studies, a 5 mm diameter agar plug was taken from an actively growing fungal colonies and placed in the centre of 90 mm diameter Petri plates containing 2% MEA. These cultures were then incubated in the dark at 5 °C intervals from 5 to 40 °C. There were five replicate plates of each strain at each temperature, and two orthogonal diameter measurements were recorded daily until the fastest-growing mycelium reached the edge of the MEA plate. Colony colors were described based on the color chart of Rayner (1970). All relevant data pertaining to type specimens were deposited into MycoBank (http://www.MycoBank.org/).

### DNA extraction, amplification, and nucleotide sequencing

Prior to DNA extraction, the strains were grown on 2% MEA at 25 °C for 5 to 7 days. The actively growing mycelium from one MEA plate per strain was scraped from the surface of the medium using a sterile scalpel and transferred to 1.5 μL Eppendorf tubes. DNA extractions and purification were conducted using an Invisorb Spin Plant Mini Kit (Tiangen, Beijing, China) following the manufacturer’s instructions. The primer pairs ITS1/ITS4 (White et al. 1990), LROR/LR5 (Vilgalys & Hester 1990), Algr52_412-433_f1/Algr52_1102_1084_r1 (Stielow et al. 2015), Bt2a/Bt2b (Glass & Donaldson 1995), EF1F/EF2R (Jacobs et al. 2004) or EF2F (Marincowitz et al. 2015)/EF2R, and CL2F/CL2R (Duong et al. 2012) or CL3F/CL3R (Musvuugwa et al. 2015) were used for amplification of internal transcribed spacer regions 1 and 2 of the nuclear ribosomal DNA operon, including the 5.8S region (ITS), the nuclear ribosomal large subunit region (LSU), the partial 60S ribosomal protein RPL10 gene (60S), the β-tubulin gene region (βT), the transcription elongation factor-1α gene region (EF-1α), and the calmodulin gene region (CAL), respectively.

The PCR assays were performed in 25 μL volumes (2.5 mM MgCl_2_, 1 × PCR buffer, 0.2 mM dNTP, 0.2 mM of each primer, and 2.5 U Taq polymerase enzyme). The PCR conditions for amplification of the ITS region were an initial denaturation step at 94 °C for 3 min, followed by 35 cycles of 1 min at 94 °C, 45 s at 55 °C, and 1 min at 72 °C, and then final chain elongation at 72 °C for 8 min. The five other gene regions were amplified using a denaturation step at 95 °C followed by 35 cycles under the same conditions as above, except that the annealing temperatures varied between 54 and 58 °C depending on the primers used, and a final chain elongation at 72 °C for 8 min. The PCR products were cleaned using a MSB Spin PCRapace Kit (250) (Invitek, Berlin, Germany) according to the manufacturer’s instructions.

Sequencing reactions were performed using a CEQ DTCS Quick Start Kit (Beckman Coulter, Brea, CA, USA) according to the manufacturer’s instructions with the same PCR primers as above. Nucleotide sequences were determined using a CEQ 2000 XL capillary automated sequencer (Beckman Coulter).

### Phylogenetic analysis

Preliminary identifications of the strains were conducted using standard BLAST searches. Representative sequences with the highest similarity matching and type strain sequences of similar species were downloaded from GenBank. Alignments were constructed with the online tool MAFFT v.7 (Katoh & Standley, 2013). The genus-level dataset was aligned using the FFT-NS-i strategy with a 200 PAM/k = 2 scoring matrix, a gap opening penalty of 1.53, and an offset value of 0.00 (Linnakoski et al. 2016). The species complex or group-level datasets consisted of closely-related DNA sequences and were thus aligned using the G-INS-i strategy with a 1 PAM/k = 2 scoring matrix, a gap opening penalty of 1.53, and an offset value of 0.00 (Linnakoski et al. 2016). Datasets were compiled in Molecular Evolutionary Genetic Analyses (MEGA) 7.0 (Kumar et al. 2016). Phylogenetic analyses of the aligned sequences were conducted using the maximum parsimony (MP), maximum likelihood (ML), and Bayesian inference (BI) methods.

PAUP* version 4.0b10 (Swofford 2003) was used for MP analysis, with gaps treated as a fifth base. One thousand bootstrap replicates were generated to estimate the branch node confidence, with max trees set to 200 and clades compatible with the 50% majority rule in the bootstrap consensus tree were retained. The analysis settings were as follows: tree bisection reconnection branch swapping, starting tree obtained via stepwise addition, steepest descent not in effect, and MulTrees effective.

ML phylogenetic analyses were conducted using RAxML-HPC v.8.2.3 (Stamatakis 2014) available in the CIPRES Science Gateway (Miller et al. 2010, http://www.phylo.org/); the GTR + G model of site substitution included estimation of Gamma-distributed rate heterogeneity and a proportion of invariant sites (Stamatakis 2006). ML bootstrap support values were estimated using 1000 bootstrap replicates.

For Bayesian analyses, the best substitution models for each data set were determined using the corrected Akaike Information Criterion (AICc) in jModelTest v. 2.1.7 (Darriba et al. 2012). Bayesian inferences using four Markov Chain Monte Carlo (MCMC) chains were run simultaneously in MrBayes v. 3.1.2 (Ronquist & Huelsenbeck 2003) from a random starting tree for 5,000,000 generations to calculate posterior probabilities. Trees were sampled every 100 generations, and the first 25% of trees sampled were discarded as burn-in, while the remaining trees were used to calculate Bayesian posterior probabilities of the clades. Phylogenetic trees were edited in FigTree v. 1.4.3 (http://tree.bio.ed.ac.uk/software/figtree/) and Adobe Illustrator CS6. The final alignments and the retrieved topologies were deposited in TreeBASE (No. 24283).

## RESULTS

### Collection of samples and isolation of fungi

In total, 496 strains of ophiostomatoid fungi were obtained from the adult beetles and galleries. Growth rates, macro- and microscopical morphological features were used for preliminary identification. Standard nucleotide BLAST searches at GenBank were performed using the BT sequences of all strains for preliminary sorting and searching for affinities. Subsequently, 41 representative strains were selected for in depth morphological study and multi-locus phylogenetic analysis (Table 1).

### Phylogenetic analysis

The three phylogenetic methods used resulted in similar topologies with slight variations of the statistical support for each of the individual sequence datasets. Phylograms obtained by ML are presented for all the individual datasets, with branch supports obtained from ML, MP, and BI analyses indicated. The best-fit evolutionary models selected by jModelTest v. 2.1.7 were GTR + G [for the CAL dataset of *O. piceae* complex, the combined datasets (βT + CAL + EF-1α) of *O. piceae* and *O. clavatum* complexes, the EF-1α datasets of *O. piceae* and *O. clavatum* complexes, the ITS dataset of *O. ips* complex], GTR + I (for the ITS datasets of *O. minus* complex and Group A, the EF-1α dataset of *Leptographium*, the LSU dataset of *Ceratocystiopsis*), GTR + I + G (for the ITS dataset of *Ophiostoma*), HKY + G (for the βT datasets of *O. piceae* and *O. minus* complexes, the CAL dataset of *O. clavatum* complex), HKY + I (for the βT datasets of *O. clavatum* and *O. ips* complexes, as well as Group A) and K80 (for the 60S dataset of *Endoconidiophora*).

**Fig. 2:**
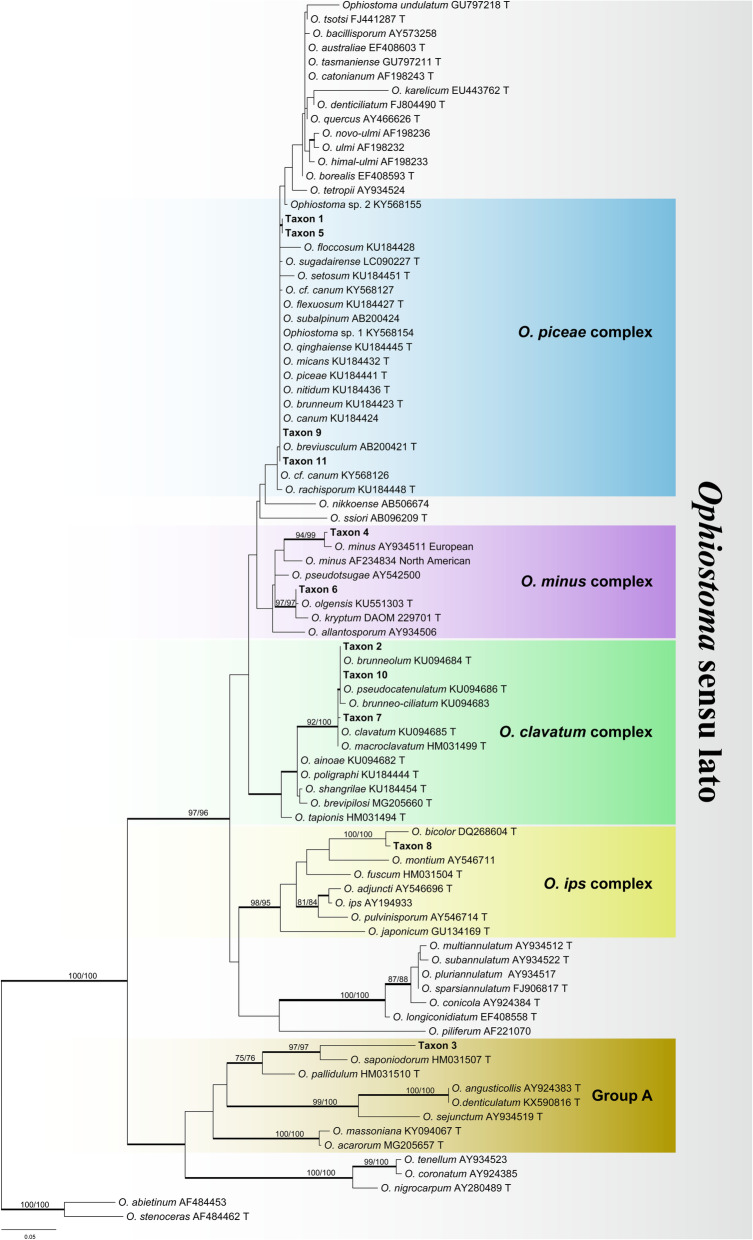
ML tree of *Ophiostoma* generated from the ITS sequence data. Sequences generated from this study are printed in bold. Bold branches indicate posterior probability values ≥0.9. Bootstrap values of ML/MP ≥ 70% are recorded at the nodes. T = ex-type isolates

**Fig. 3:**
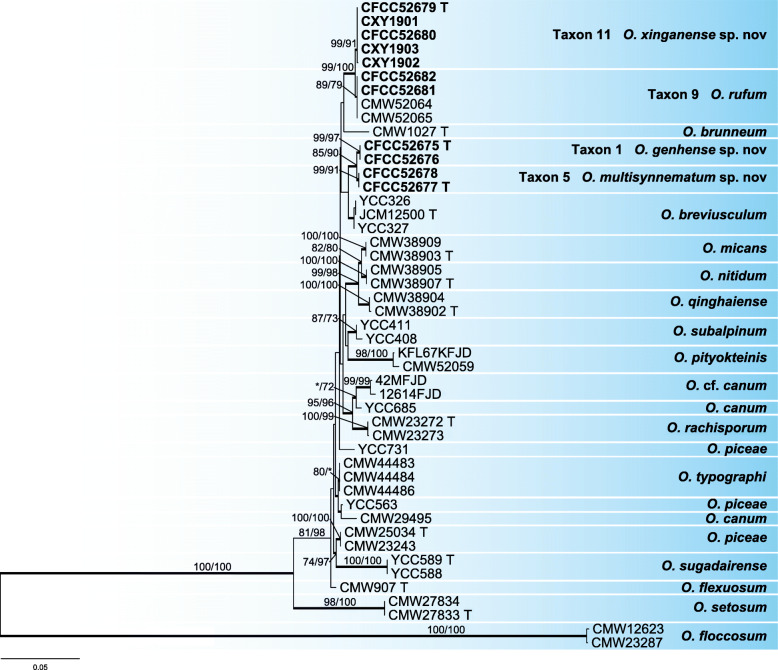
ML tree of *O. piceae* complex generated from the combined (βT + CAL + EF-1α) sequence data. Sequences generated from this study are printed in bold. Bold branches indicate posterior probability values ≥0.9. Bootstrap values of ML/MP ≥ 70% are recorded at the nodes. T = ex-type isolates

The ITS sequences did not allow distinguishing closely related species in all cases but they enabled grouping strains into species complexes within *Ophiostoma* (Fig. 2). However, the partial DNA sequences of three protein-coding genes (βT, CAL, EF-1α, and combined) had sufficient internal information allowing identification of *Ophiostoma* to the species level (Figs. 3, 5, 6, 8, 10; Additional file 2: Figure S1, Additional file 3: Figure S2, Additional file 4: Figure S3, Additional file 5: Figure S4, Additional file 6: Figure S5, Additional file 7: Figure S6). The LSU, 60S, or EF DNA sequences also were employed to identify the strains of other three genera (*Ceratocystiopsis*, *Endoconidiophora*, and *Leptographium*) (Fig. 11; Additional file 8: Figure S7, Additional file 9: Figure S8).

The phylogenetic analyses showed that our 41 representative strains belonged to 14 terminal clades or phylogenetic species. Eleven phylogenetic species nested within the *Ophiostoma* lineage (Taxa 1–11), whereas the last three belonged to the *Ceratocystiopsis* (Taxon 12), *Leptographium* (Taxon 13), and *Endoconidiophora* lineages (Taxon 14).

### *Ophiostoma* s. l.

Within *Ophiostoma* s.l., the ITS fragments were approximately 650 bp long. The ITS dataset included 88 entries representing 85 taxa and 663 positions (including gaps). Our strains nested in four species complexes in phylogenetic inferences, viz. the *O. piceae* complex (four representative strains), the *O. minus* complex (two representative strains), the *O. clavatum* complex (three representative strains), and the *O. ips* complex (one representative strain) (Fig. 2). Furthermore, one strains (representing one phylogenetic species) fall outside any currently recognized species complex but belong to the previously shown “Group A” (Chang et al. 2017).

Several DNA sequences subsets were compiled for identification of species level within species complexes. Three ITS subsets for the *O. minus* and *O. ips* complexes, and group A comprised respectively of 589, 652 and 554 characters; five βT subsets for the *O. piceae*, *O. minus*, *O. clavatum*, and *O. ips* complexes, as well as group A, contained 403, 442, 430, 441, and 565 characters, respectively; two EF-1α subsets for the *O. piceae* and *O. clavatum* complexes, contained 1052 and 977 characters, respectively; two CAL subsets for the *O. piceae* and *O. clavatum* complexes, consisted of 961 and 931 characters, respectively. Two combined datasets (βT + CAL + EF-1α) for the *O. piceae* and *O. clavatum* complexes, consisted of 2418 and 2340 characters, respectively, including gaps.

Our 11 representative strains within the *O. piceae* complex formed four independent well-supported terminal clades representing four phylogenetic species (Taxa 1, 5, 9, and 11) in combined datasets (βT + CAL + EF-1α) phylogenetic inferences (Fig. 3). These phylogenetic species were related to *O. breviusculum*, *O. brunneum*, and *O. rufum* (Jankowiak et al. 2019). Clades of taxa 1 and 5 are well-supported in phylogenetic analyses based on the βT, EF-1α, CAL, and combined datasets (Additional file 2: Figure S1, Additional file 3: Figure S2, Additional file 4: Figure S3, Fig. 3). Clades of taxa 9 and 11 are shown in the EF-1α-based (Additional file 3: Figure S2) and combined datasets (βT + CAL + EF-1α) for the *O. piceae* complex phylogenetic analyses (Fig. 3), whereas in phylogenetic inferences based on βT and CAL, these two clades collapsed (Additional file 2: Figure S1, Additional file 4: Figure S3).

**Fig. 4:**
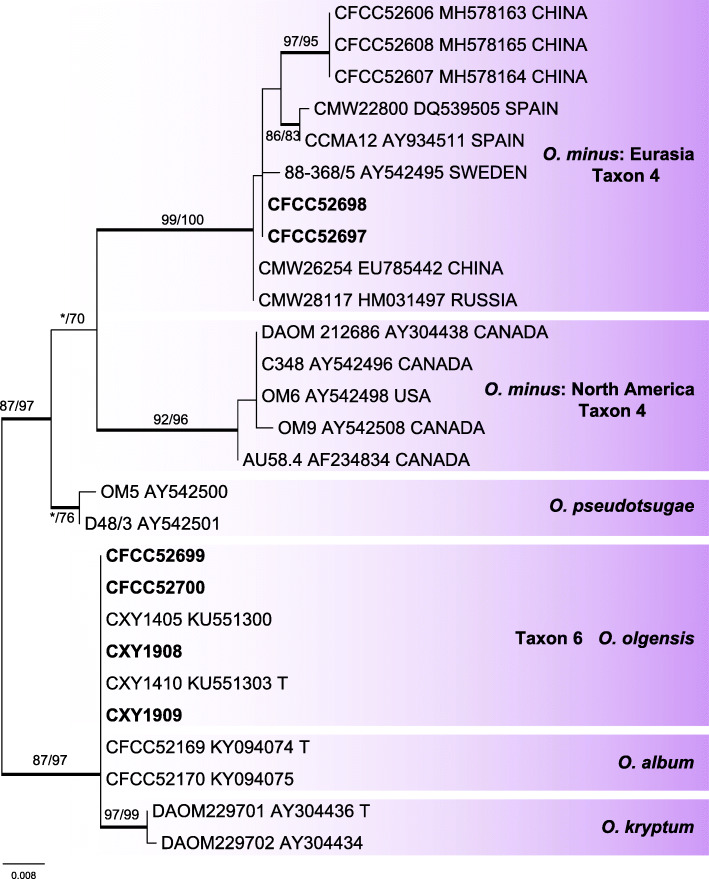
ML tree of *O. minus* complex generated from the ITS sequence data. Sequences generated from this study are printed in bold. Bold branches indicate posterior probability values ≥0.9. Bootstrap values of ML/MP ≥ 70% are recorded at the nodes. T = ex-type isolates

**Fig. 5:**
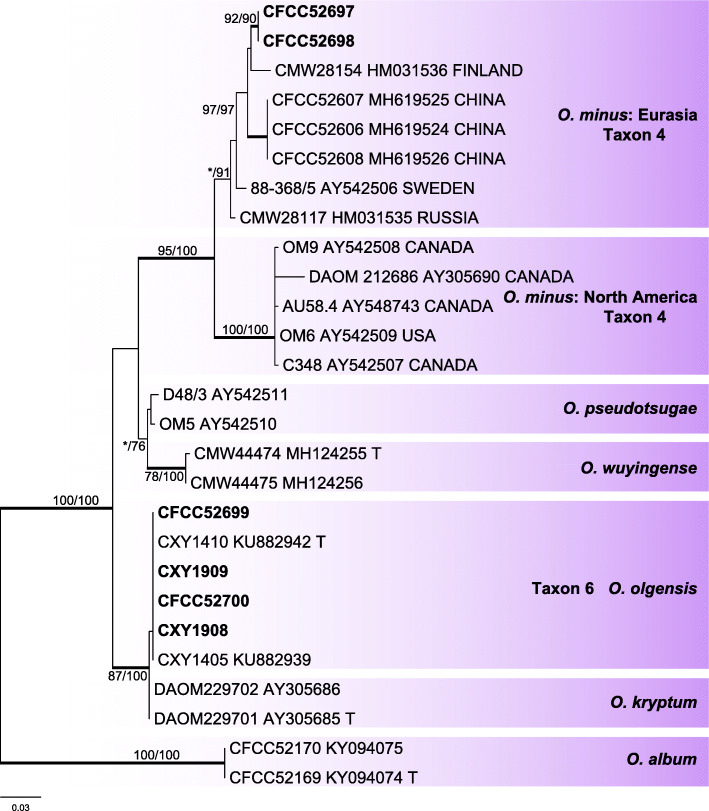
ML tree of *O. minus* complex generated from the βT sequence data. Sequences generated from this study are printed in bold. Bold branches indicate posterior probability values ≥0.9. Bootstrap values of ML/MP ≥ 70% are recorded at the nodes. T = ex-type isolates

Six representative strains of the *O. minus* complex were grouped into two independent well-supported clades of taxa 4 and 6 in the ITS based inferences (Fig. 4). The clade of taxon 4 was related to the *O. minus* Eurasian clade (Gorton & Webber 2000, Lu et al. 2009), and the clade of taxon 6 nested in the near vicinity of the *O. olgensis* and *O. album* clades. In the βT-based phylogram, the clade of taxon 4 separated into a well-supported subclade, which is a sister to a formerly defined Eurasian subclade within the *O. minus* clade (Fig. 5). Furthermore, the βT-based phylogram confirmed the strains of clade of taxon 6 represented *O. olgensis* (Fig. 5).

**Fig. 6:**
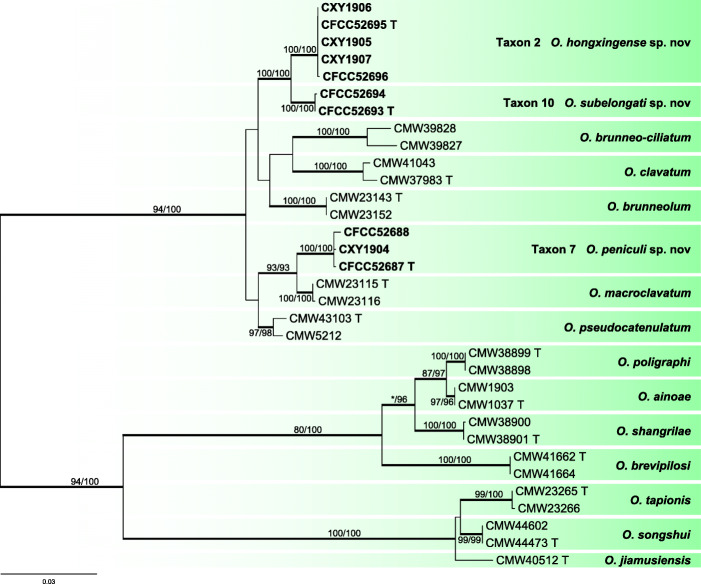
ML tree of *O. clavatum* complex generated from the combined (βT + CAL + EF-1α) sequence data. Sequences generated from this study are printed in bold. Bold branches indicate posterior probability values ≥0.9. Bootstrap values of ML/MP ≥ 70% are recorded at the nodes. T = ex-type isolates

Ten representative strains belonging to the *O. clavatum* complex formed three independent terminal clades (Taxa 2, 7, and 10) with good support values in inferences based on the βT (Additional file 5: Figure S4), EF-1α (Additional file 6: Figure S5), CAL (Additional file 7: Figure S6), and combined dataset (βT + CAL + EF-1α) (Fig. 6). They were related to the *O. brunneo-ciliatum*, *O. brunneolum*, *O. clavatum*, *O. macroclavatum* and *O. pseudocatenulatum* species clades.

**Fig. 7:**
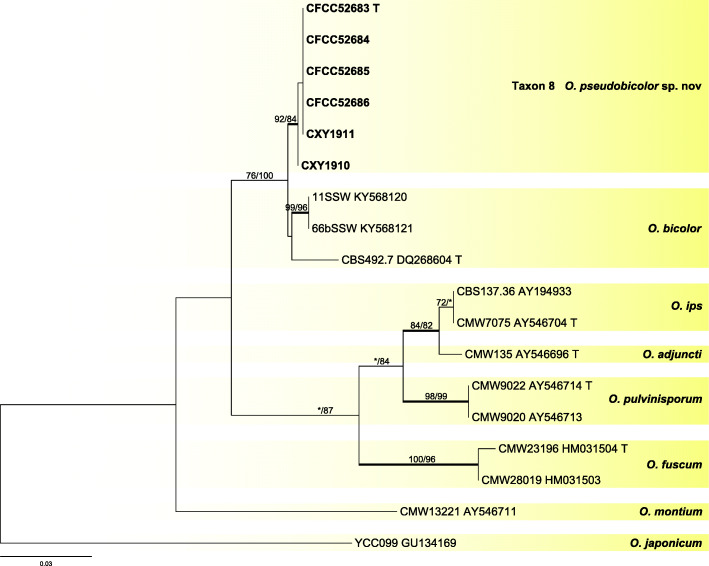
ML tree of *O. ips* complex generated from the ITS sequence data. Sequences generated from this study are printed in bold. Bold branches indicate posterior probability values ≥0.9. Bootstrap values of ML/MP ≥ 70% are recorded at the nodes. T = ex-type isolates

**Fig. 8:**
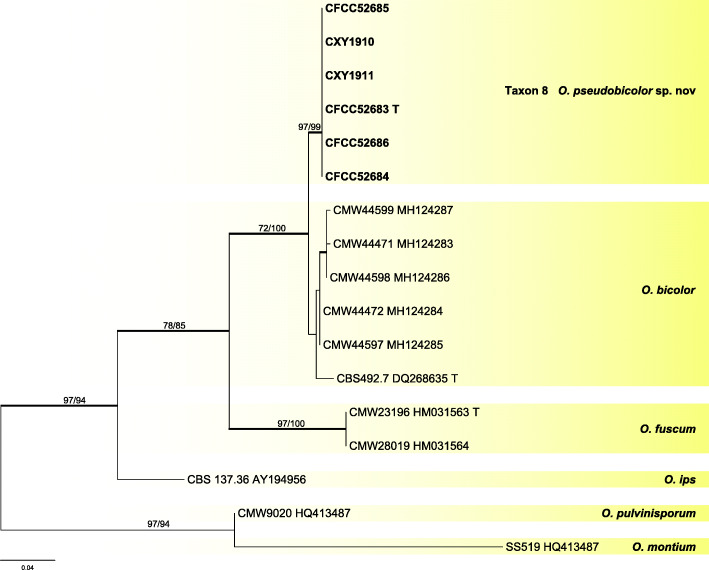
ML tree of *O. ips* complex generated from the βT sequence data. Sequences generated from this study are printed in bold. Bold branches indicate posterior probability values ≥0.9. Bootstrap values of ML/MP ≥ 70% are recorded at nodes. T = ex-type isolates

Six representative strains of taxon 8 in the *O. ips* complex were used in the analyses. Taxon 8 formed a distinct clade with good statistical support in ITS (Fig. 7) and βT (Fig. 8) analyses. It was closely related to *O. bicolor*.

**Fig. 9:**
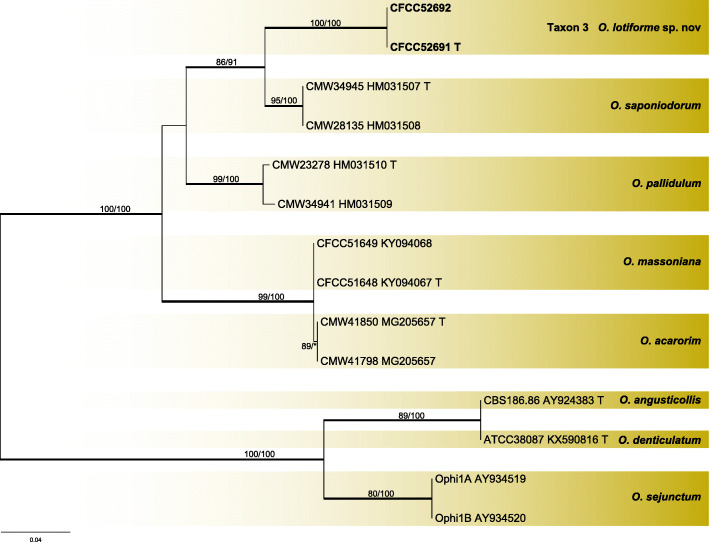
ML tree of Group A generated from the ITS sequence data. Sequences generated from this study are printed in bold. Bold branches indicate posterior probability values ≥0.9. Bootstrap values of ML/MP ≥ 70% are recorded at the nodes. T = ex-type isolates

**Fig. 10:**
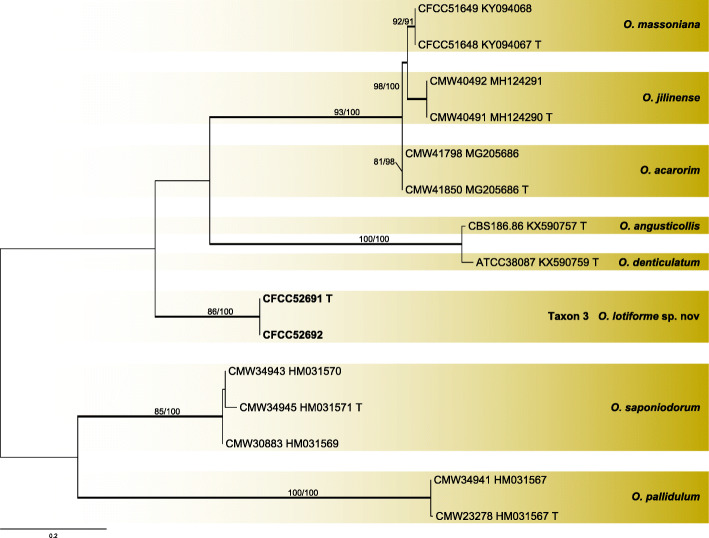
ML tree of Group A generated from the βT sequence data. Sequences generated from this study are printed in bold. Bold branches indicate posterior probability values ≥0.9. Bootstrap values of ML/MP ≥ 70% are recorded at the nodes. T = ex-type isolates

Two strains of taxon 3 in Group A were used in the analyses. Taxon 3 formed an independent lineage with good support values in ITS and βT based phylograms (Figs. 9, 10) and was most closely related to *O. saponiodorum* and *O. pallidulum*.

### *Ceratocystiopsis*

**Fig. 11:**
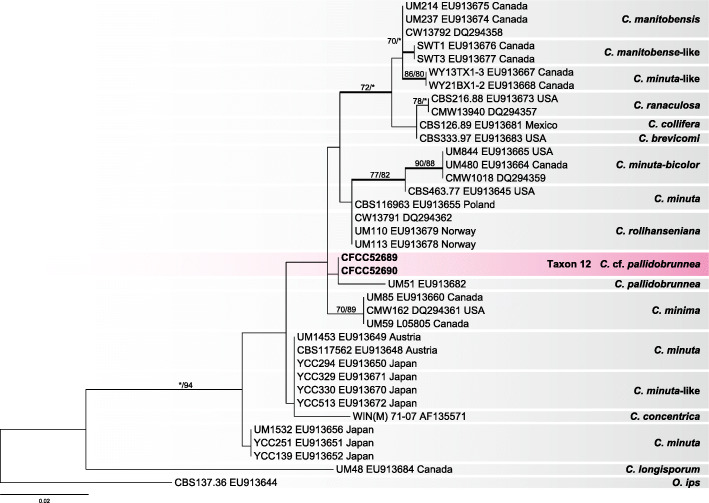
ML tree of *Ceratocystiopsis* generated from the LSU sequence data. Sequences generated from this study are printed in bold. Bold branches indicate posterior probability values ≥0.9. Bootstrap values of ML/MP ≥ 70% are recorded at the nodes

The LSU phylogenetic tree of the genus *Ceratocystiopsis* did not yield an independent clade for taxon 12 (Fig. 11), although its strains showed clear dissimilarity with *C. pallidobrunnea* in terms of the LSU sequence data. The lack of available reference data in GenBank for other genes for *C. pallidobrunnea* impeded any closer comparison. Therefore, this species is *hitherto* recorded as *Ceratocystiopsis* cf. *pallidobrunnea*.

### *Leptographium* and *Endoconidiophora*

Sequence comparisons and phylogenetic analyses revealed that representative strains of taxa 13 and 14 had identical sequences respectively and clustered into the same clades as *Leptographium zhangii* and *Endoconidiophora fujiensis* based on EF-1α and 60S genes (Additional file 8: Figure S7, Additional file 9: Figure S8)*.* Therefore, these two phylogenetic species were defined as the known species previously discovered in China (Meng et al. 2015, Liu et al. 2017).

## TAXONOMY

Eight of 14 phylogenetic species identified in this study were shown to represent distinct terminal clades, and are interpreted as new species of *Ophiostoma*.

***Ophiostoma genhense*** Z. Wang & Q. Lu, **sp. nov.**

MycoBank MB 830610.

(Fig. 12)

**Fig. 12:**
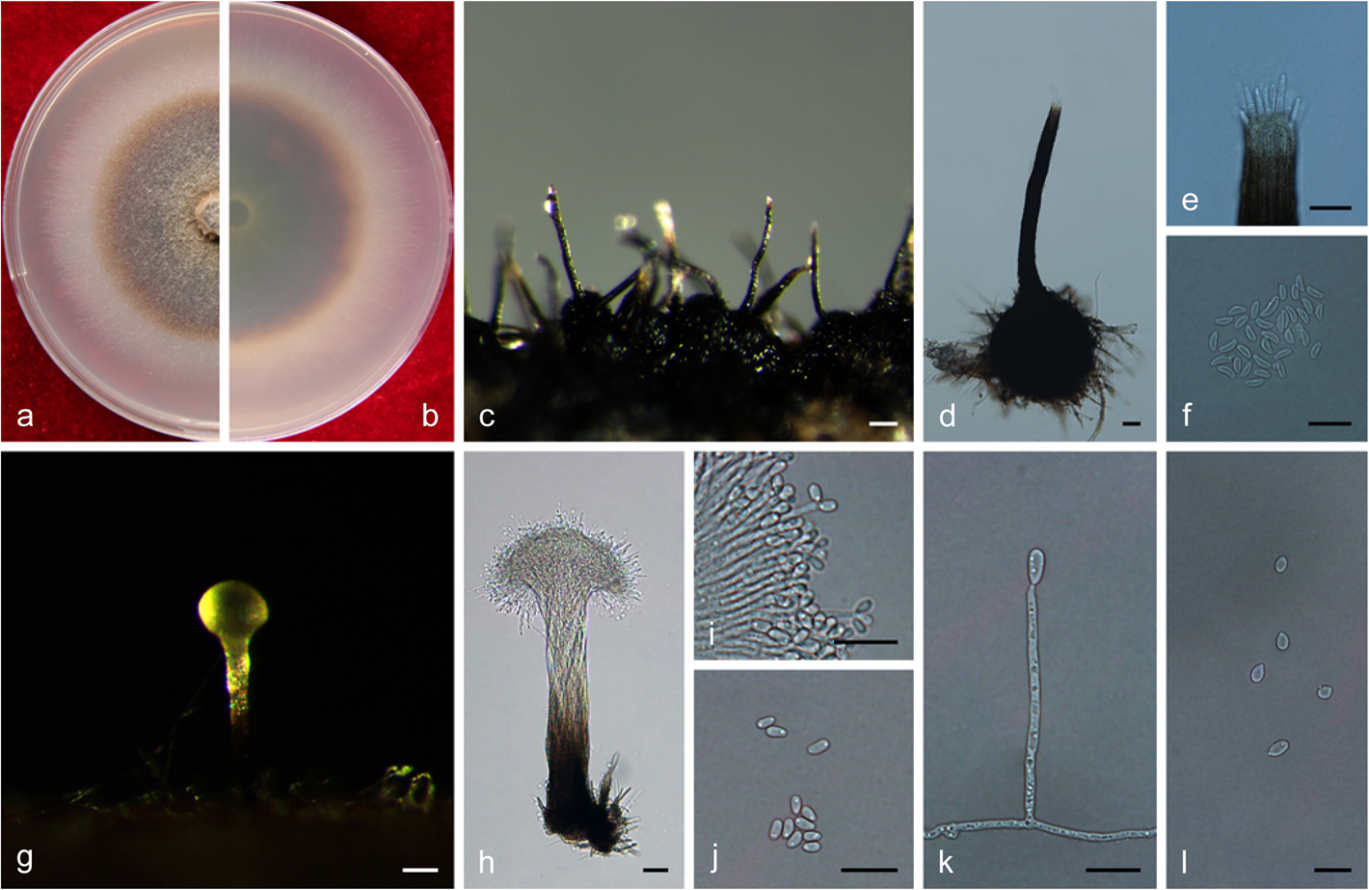
Morphological characteristics of *Ophiostoma genhense* sp. nov. (CFCC 52675, Taxon 1). **a–b.** Ten-day-old cultures on 2% MEA; **c–d.** Perithecium. **e.** Ostiolar hyphae. **f.** Ascospores. **g–h.** Pesotum-like asexual morph; **i–j.** Conidiogenous cells of pesotum-like asexual morph and conidia; **k–l.** Hyalorhinocladiella-like asexual morph: conidiogenous cells and conidia. Scale bars: c = 50 μm; d = 20 μm; e–f = 10 μm; g = 50 μm; h = 20 μm; i–l = 10 μm

*Etymology*: The epithet *genhense* (Latin) refers to the city of Genhe, from which this fungus was collected.

*Diagnosis*: The species is characterized by perithecia and synnematous conidiophores. It can be differentiated from the closely related species *O. multisynnematum* by the presence of perithecia, absent in latter, and smaller synnemata. Over time, the *O. genhense* colonies gradually turned brown from the centre, whereas the colonies of *O. multisynnematum* turned dark olivaceous.

*Type*: **China**, *Inner Mongolia Autonomous Region*: Genhe, from *Ips subelongatus* infesting *Larix gmelinii*, Sept. 2017, *Q. Lu* (CXY 2001 – holotype; CFCC 52675 – ex-type culture).

*Description*: *Sexual morph* perithecial. *Perithecia* few on 2% MEA after 20 d, developing on a superficial mycelium or partly embedded in the agar, bases black, (103–) 114–156 (− 164) μm diam., with some basal hyphal ornamentation, dark brown to black; necks black, cylindrical, straight or slightly curved, (135–) 210–347 (− 400) μm long, (17–) 21.5–32.5 (− 38) μm wide at the base down to (8.5–) 12.5–17 (− 18.5) μm wide at the apex, composed of parallel, septate, laterally fused hyphae, ending in a crown of hyaline. *Ostiolar hyphae* occasionally present, 6.5–13 (− 18) μm long. *Ascospores* hyaline, allantoid or crescent in side view, without sheath, aseptate, (3.5–) 4–5 (− 6) × (1.5–) 2 (− 2.5) μm.

*Asexual morphs*: pesotum-like and hyalorhinocladiella-like.

*Pesotum-like morph*: *synnemata* solitary or in groups, the base black, (22.5–) 24.5–45.5 (− 48.5) μm wide, (170–) 184–257 (− 271) μm tall, including the conidiogenous apparatus. *Conidiogenous cells* (12–) 15–23 (− 26.5) × 1.5–2 μm. *Conidia* hyaline, smooth, cylindrical, aseptate, (3–) 3.5–4 (− 4.5) × 2–2.5 μm. *Hyalorhinocladiella-like morph*: *conidiogenous cells* arising directly from the hyphae, (25–) 30.5–43 (− 44) × 1.5–2 μm. *Conidia* hyaline, smooth, ovate to cylindrical, aseptate, (3.5–) 4–5.5 (− 7) × (2.5–) 3–3.5 (− 4) μm.

*Cultures*: *Colonies* on 2% MEA at 25 °C reaching 80 mm diam. in 10 d, initially hyaline, later becoming brown, mycelium superficial or sparsely aerial, the colonies edge thinning radially, synnemata and perithecia scattered in the centre. Optimal temperature for growth at 25 °C, no growth observed at 5 °C and 35 °C.

*Ecology*: Isolated from *Ips subelongatus* infesting dying *Larix gmelinii* and stock log.

Habitat: *L. gmelinii* pure plantation.

*Distribution*: Currently only known from the Inner Mongolia Autonomous Region, China.

*Notes*: *Ophiostoma genhense* and *O. multisynnematum* formed two distinct, well-supported clades within the *O. piceae* complex (Fig. 3), in which they were closely related to *O. breviusculum* (Chung et al. 2006). They can be both differentiated from *O. breviusculum* by the presence of a hyalorhinocladiella-like asexual state, which is absent in the latter.

*Additional specimens examined*: **China**: *Inner Mongolia Autonomous Region*: Genhe, from *Ips subelongatus* infesting *Larix gmelinii*, Sept. 2017, *Q. Lu* (cultures CXY 2002, CFCC 52676).

***Ophiostoma hongxingense*** Z. Wang & Q. Lu, **sp. nov.**

MycoBank MB 830611.

(Fig. 13)

**Fig. 13:**
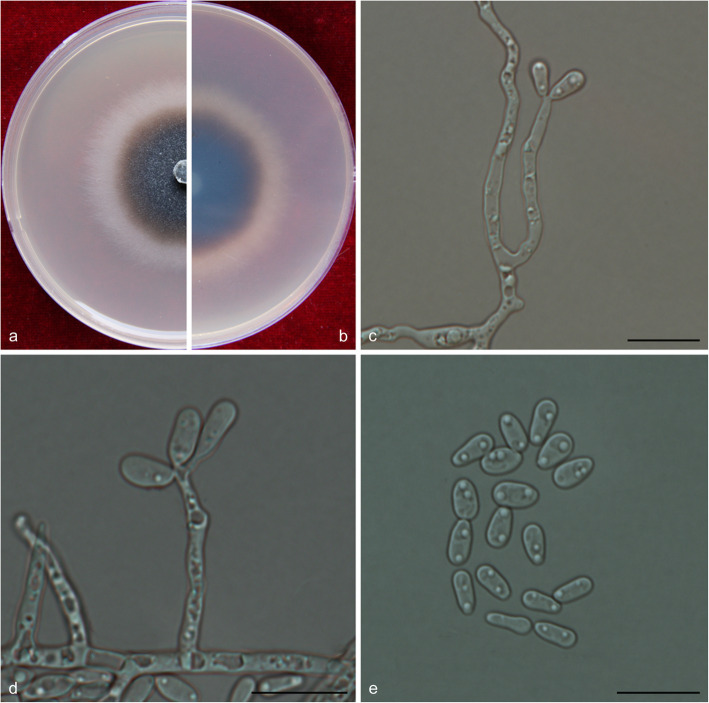
Morphological characteristics of *Ophiostoma hongxingense* sp. nov. (CFCC 52695, Taxon 2). **a–b.** Five-day-old cultures on 2% MEA; **c–e.** Hyalorhinocladiella-like asexual morph: conidiogenous cells and conidia. Scale bars: c–e = 10 μm

*Etymology*: The epithet *hongxingense* (Latin) refers to the city of Hongxing, from which this fungus was collected.

*Diagnosis*: See comparisons between *Ophiostoma hongxingense* and *O. subelongati* under *O. subelongati*.

*Type*: **China**: *Heilongjiang province*: Hongxing, from *Ips subelongatus* on *Larix gmelinii*, July 2017, *Q. Lu* (CXY 2021 – holotype; CFCC 52695 – ex-type culture).

*Description*: *Sexual morph* not observed.

*Asexual morph*: hyalorhinocladiella-like.

*Hyalorhinocladiella-like morph*: *conidiogenous cells* arising directly from superficial hyphae, (10.5–) 14–22.5 (− 28.5) × (1.5–) 2–2.5 (− 3) μm. *Conidia* hyaline, smooth, ovate to elliptical, aseptate, (5–) 5.5–6.5 (− 8) × (2–) 2.5–3.5 (− 4) μm.

*Cultures*: *Colonies* on 2% MEA at 25 °C reaching 58 mm diam. in 5 d, initially hyaline, discoloring progressively to dark olivaceous from the centre of the colonies to the margin, the colonies edge thinning radially; mycelium superficial on the agar. Optimal temperature for growth at 30 °C, no growth observed at 5 °C and 40 °C.

*Ecology*: Isolated from *Ips subelongatus* infesting dying *Larix gmelinii* and *L. olgensis*.

Habitat: *L. gmelinii* or *L. olgensis* pure plantation.

*Distribution*: Currently known from the Inner Mongolia Autonomous Region and Heilongjiang province, China.

*Notes*: See comparisons between *Ophiostoma hongxingense* and *O. subelongati* under *O. subelongati*.

*Additional specimens examined*: **China**: *Heilongjiang province*, Zhanhe, from *Ips subelongatus* infesting *Larix gmelinii*, July 2017, *Q. Lu* (cultures CXY 2022 = CFCC 52696; CXY 1905; CXY 1906; CXY 1907); Jiamusi, from *Ips subelongatus* infesting *Larix olgensis*. Aug. 2011, *Q. Lu* (culture CXY 1924).

***Ophiostoma lotiforme*** Z. Wang & Q. Lu, **sp. nov.**

MycoBank MB 830612.

(Fig. 14)

**Fig. 14:**
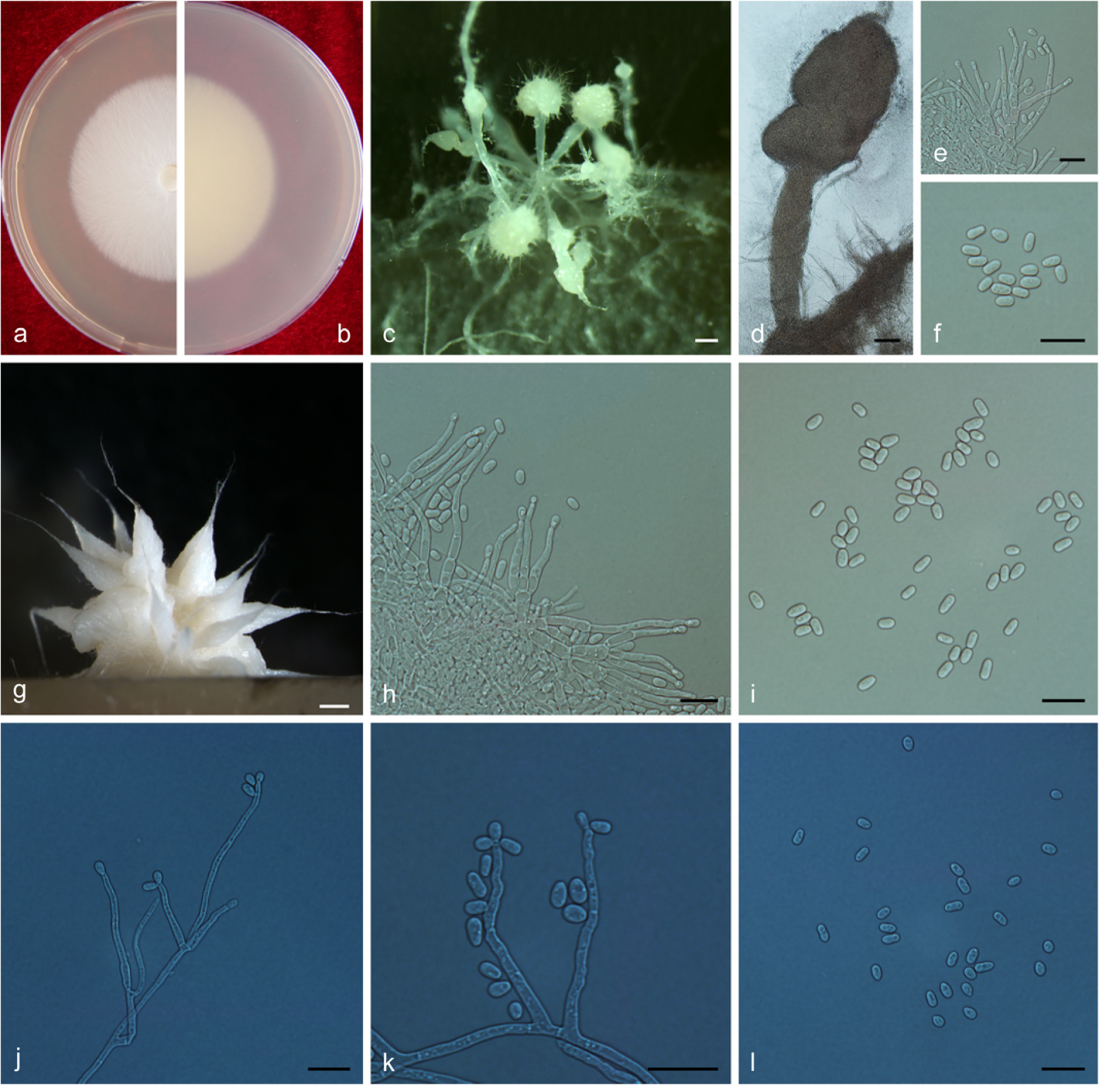
Morphological characteristics of *Ophiostoma lotiforme* sp. nov. (CFCC 52691, Taxon 3). **a–b.** Fifteen-day-old cultures on 2% MEA; **c–d.** Pesotum-like asexual morph; **e–f.** Conidiogenous cells of pesotum-like asexual morph and conidia; **g.** Lotus-shaped conidiomata; **h–i.** Conidiogenous cells of lotus-shaped conidiomata and conidia; **j–l.** Hyalorhinocladiella-like asexual morph: conidiogenous cells and conidia. Scale bars: c = 200 μm; d = 100 μm; e–f = 10 μm, g = 200 μm; h–l = 10 μm

*Etymology*: The epithet *lotiforme* (Latin) refers to lotus-shaped conidiomata composed of clustered synnemata.

*Diagnosis*: The compound, lotus-shaped conidiomata are unique, and its synnemata are distinctly taller than those of the related species *O. saponiodorum*, respectively (876–) 945–1224 (− 1290) μm vs. (118–) 188–297 (− 370) μm. Synnemata are absent in *O. pallidulum* (Linnakoski et al. 2010). *Ophiostoma lotiforme* also grows slower than *O. saponiodorum* on 2% MEA. In addition, no growth of *O. saponiodorum* is observed at 35 °C, but *O. lotiforme* grows at 35 °C; no growth of *O. lotiforme* is observed at 40 °C, but *O. pallidulum* grows at 40 °C.

*Type*: **China**: *Inner Mongolia Autonomous Region*, Hailar, from *Ips subelongatus* on *Pinus sylvestris* var. *mongolica*, Aug. 2010, *X. Meng* (CXY 2017 – holotype, CFCC 52691 = MUCL 55165 – ex-type culture).

*Description*: *Sexual morph* not observed.

*Asexual morphs*: pesotum-like and hyalorhinocladiella-like.

*Pesotum-like morph*: *synnemata* occurring in groups, the base hyaline, (78.5–) 81.5–91.5 (− 94) μm wide, (876–) 945–1224 (− 1290) μm tall including the conidiogenous apparatus. *Conidiogenous cells* (12–) 15–23 (− 28.5) × (1.5–) 2–2.5 μm. *Conidia* hyaline, smooth, clavate to ovate, aseptate, 4–5.5 (− 6) × 2–2.5 μm; compound, lotus–shaped, pesotum-like conidiomata, pure white, (898–) 971–1296 (− 1450) μm wide at base, (964–) 1019–1427 (− 1655) μm tall. *Hyalorhinocladiella-like morph*: *conidiogenous cells* arising from superficial hyphae, (6–) 11–22 (− 28.5) × 1.5–2.5 (− 3) μm. *Conidia* hyaline, aseptate, smooth, clavate to ovate, (3.5–) 4–5.5 (− 6.5) × (2–) 2.5–3.5 (− 4) μm.

*Cultures*: *Colonies* on 2% MEA at 25 °C reaching 65 mm diam. in 15 d, pure white, the colonies margin smooth; mycelium superficial on the agar. Optimal temperature for growth at 30 °C, no growth observed at 5 °C and 40 °C.

*Ecology*: Isolated from *Ips subelongatus* infesting dying *Pinus sylvestris* var. *mongolica*.

Habitat: Mixed forest of *P. sylvestris* var. *mongolica* and *L. gmelinii*.

*Distribution*: Currently only known from the Inner Mongolia Autonomous Region, China.

*Notes*: *Ophiostoma lotiforme* pertains to the *O. saponiodorum* lineage, in which it is closely related to *O. saponiodorum* and *O. pallidulum* (Figs. 2, 9, and 10). These species share a hyalorhinocladiella-like asexual state (Linnakoski et al. 2010).

*Additional specimens examined*: **China**: *Inner Mongolia Autonomous Region*, Hailar, from *Ips subelongatus* infesting *Pinus sylvestris* var. *mongolica*, Aug. 2010, *X. Meng* (cultures CXY 2018 = CFCC 52692).

***Ophiostoma multisynnematum*** Z. Wang & Q. Lu, **sp. nov.**

MycoBank MB 830614.

(Fig. 15)

**Fig. 15:**
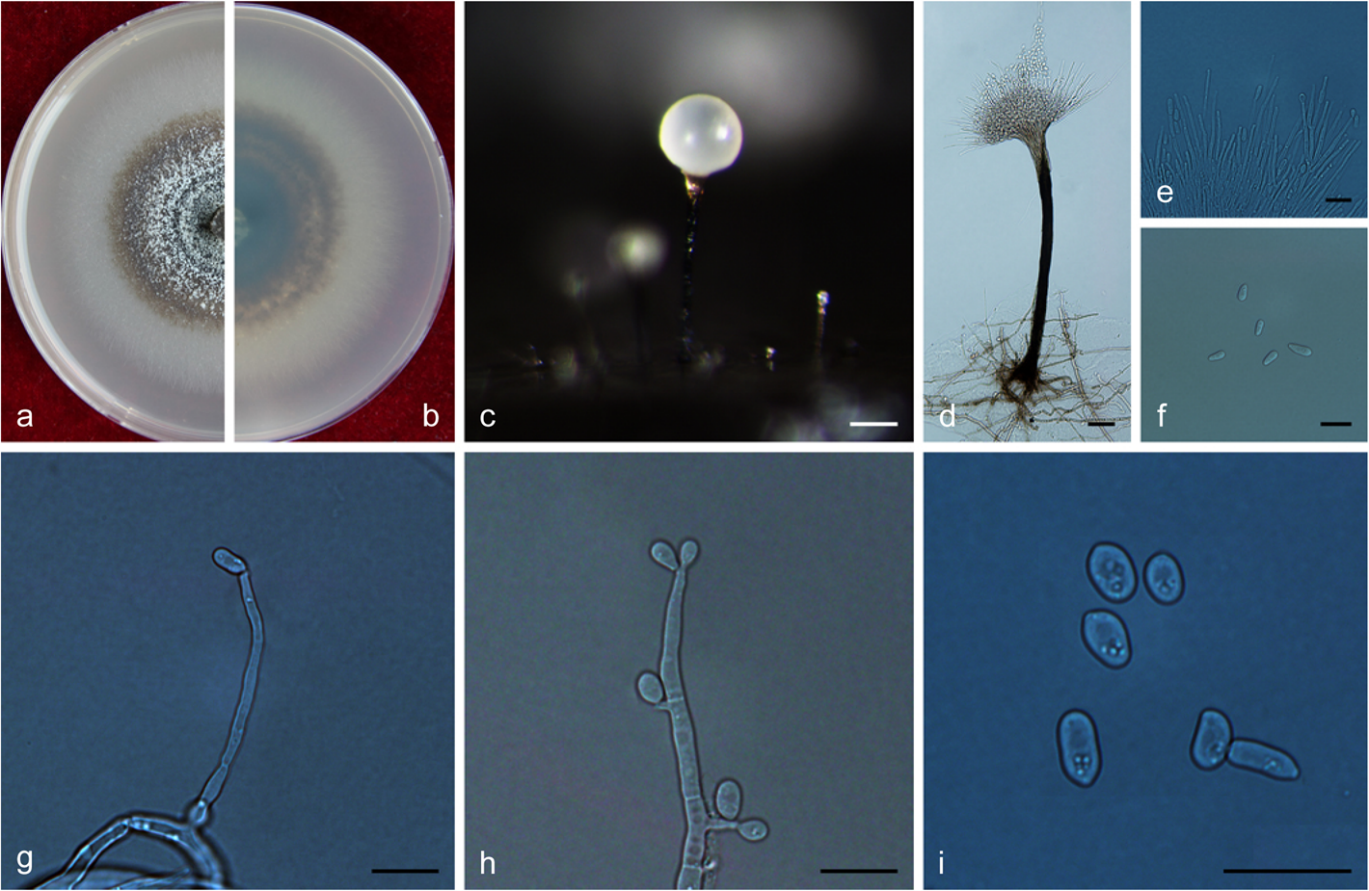
Morphological characteristics of *Ophiostoma multisynnematum* sp. nov. (CFCC 52677, Taxon 5). **a–b.** Ten-day-old cultures on 2% MEA; **c–d.** Pesotum-like asexual morph; **e–f.** Conidiogenous cells of pesotum-like asexual morph and conidia; **g–i.** Hyalorhinocladiella-like asexual morph: conidiogenous cells and conidia. Scale bars: c = 50 μm; d = 20 μm; e–i = 10 μm

*Etymology*: The epithet *multisynnematum* (Latin) referring to the numerous synnemata.

*Diagnosis*: See comparisons among *Ophiostoma multisynnematum*, *O. genhense*, and *O. breviusculum* below the description of *O. genhense*.

*Type*: **China**: *Inner Mongolia Autonomous Region*, Genhe, from *Ips subelongatus* infesting *Larix gmelinii*, Sept. 2017, *Q. Lu* (CXY 2003 – holotype; CFCC 52677 – ex-type culture).

*Description*: *Sexual morph* not observed.

*Asexual morphs*: pesotum-like and hyalorhinocladiella-like.

*Pesotum-like morph*: *synnemata* occurring singly or in groups, the base black, (11–) 12.5–43.5 (− 73) μm wide, (256–) 307–433 (− 544) μm tall including conidiogenous apparatus. *Conidiogenous cells* (12–) 17.5–31.5 (− 45) × 1.5–2 (− 2.5) μm. *Conidia* hyaline, smooth, cylindrical, aseptate, 5.5–7 (− 8.5) × (2–) 2.5–3 (− 3.5) μm. *Hyalorhinocladiella-like morph*: *conidiogenous cells* arising from superficial hyphae, (9–) 13–33.5 (− 50.5) × (1.5–) 2–2.5 (− 3) μm. *Conidia* hyaline, smooth, ovate to cylindrical, aseptate, (4–) 4.5–5.5 (− 6.5) × 2.5–3.5 (− 4.5) μm.

*Cultures*: *Colonies* on 2% MEA at 25 °C reaching 78 mm in diameter in 10 d, initially hyaline, thinning radially toward the margin, later becoming dark olivaceous and massive synnemata arising in the centre; hyphae superficial, aerial mycelium sparse. Optimal temperature for growth at 25 °C, no growth observed at 5 °C and 35 °C.

*Ecology*: Isolated from *Ips subelongatus* infesting dying *Larix gmelinii* and stock log.

Habitat: *L. gmelinii* pure plantation.

*Distribution*: Currently only known from the Inner Mongolia Autonomous Region, China.

*Notes*: See comparisons among *Ophiostoma multisynnematum*, *O. genhense*, and *O. breviusculum* below the description of *O. genhense*.

*Additional specimens examined*: **China**: *Inner Mongolia Autonomous Region*, Genhe, from *Ips subelongatus* infesting *Larix gmelinii*, Sept. 2017, *Q. Lu* (cultures CXY 2004 = CFCC 52678; CXY 1917; CXY 1918; CXY 1919).

***Ophiostoma peniculi*** Z. Wang & Q. Lu, **sp. nov.**

MycoBank MB 830609.

(Fig. 16)

**Fig. 16:**
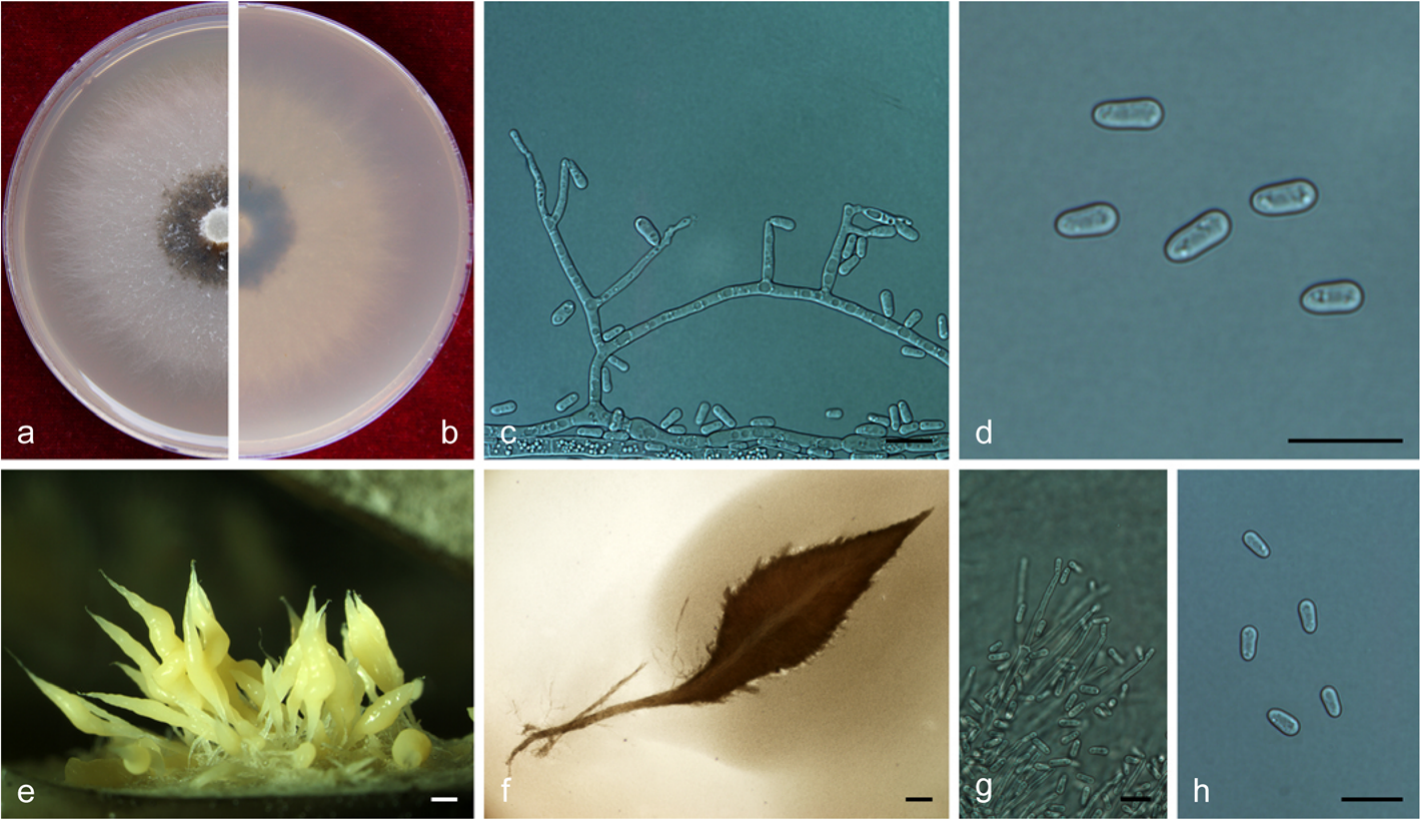
Morphological characteristics of *Ophiostoma peniculi* sp. nov. (CFCC 52687, Taxon 7). **a–b.** Five-day-old cultures on 2% MEA; **c–d.** Hyalorhinocladiella-like asexual morph: conidiogenous cells and conidia. **e.** Brush-like conidioma; **f.** Pesotum-like asexual morph; **g–h.** Conidiogenous cells of pesotum-like asexual morph and conidia. Scale bars: c–d = 10 μm; e = 400 μm; f = 200 μm; g–h = 10 μm

*Etymology*: The epithet *peniculi* (Latin) refers to the brush-like conidiomata.

*Diagnosis*: *Ophiostoma peniculi*, *O. macroclavatum*, and *O. pseudocatenulatum* can be distinguished from each other by the sizes of their synnemata and conidia. In decreasing order, the size ranges of their synnemata are (2184–) 3117–5172 (− 6330) μm in *O. macroclavatum*, (1366–) 1931–3696 (− 4534) μm in *O. pseudocatenulatum* and (875) 945–1224 (− 1290) μm in *O. peniculi*. The width ranges of their conidia are, in decreasing order, 2–2.5 μm in *O. peniculi*, 1.5–2 (− 3) μm in *O. macroclavatum*, and (0.5–) 1–1.5 (− 2) μm in *O. pseudocatenulatum*. *Ophiostoma peniculi* colonies also grow faster than the above two species on 2% MEA at 25 °C. The optimal growth temperature is also different for *O. peniculi* (30 °C) and *O. macroclavatum* (25 °C). As for *O. peniculi*, no growth was observed at 5 °C and 40 °C, but *O. pseudocatenulatum* can still grow at 5 °C (Linnakoski et al. 2016).

*Type*: **China**: *Heilongjiang province*, Hongxing, from *Ips subelongatus* infesting *Larix gmelinii*, Sept. 2017, *Q. Lu* (CXY 2013 – holotype, CFCC 52687 – ex-type culture).

*Description*: *Sexual morph* not observed.

*Asexual morphs*: pesotum-like and hyalorhinocladiella-like.

*Pesotum-like morph*: *synnemata* brush-like, occurring in groups, milky white, (875–) 945–1225 (− 1290) μm long including conidiogenous apparatus, (78.5–) 81.5–91.5 (− 94) μm wide at base. *Conidiogenous cells* (13.5–) 17.5–26.5 (− 32) × (1.5–) 2–2.5 μm. *Conidia* hyaline, smooth, cylindrical, aseptate, (4–) 5–6 (− 6.5) × 2–2.5 μm. *Hyalorhinocladiella-like morph*: *conidiogenous cells* arising directly from aerial hyphae, (7.5–) 10.5–21 (− 33.5) × (1.5–) 2–2.5 (− 3) μm. *Conidia* hyaline, aseptate, smooth, cylindrical, (5–) 5.5–6.5 (− 8) × (28–) 2.5–3.5 (− 4) μm.

*Cultures*: *Colonies* on 2% MEA at 25 °C reaching 75 mm diam. in 5 d, initially hyaline, the colonies edge thinning radially, becoming dark olivaceous in the centre; mycelium mostly superficial, sparsely aerial. Optimal temperature for growth at 30 °C, no growth observed at 5 °C and 40 °C.

*Ecology*: Isolated from *Ips subelongatus* infesting dying *Larix gmelinii* and *L. olgensis*.

Habitat: *L. gmelinii* or *L. olgensis* pure plantation.

*Distribution*: Currently known from the Inner Mongolia Autonomous Region and Heilongjiang province, China.

*Notes*: In a phylogenetic perspective, *O. peniculi* is closely related to *O. hongxingense*, *O. subelongati*, *O. macroclavatum*, *O. pseudocatenulatum*, and *O. brunneolum* (Fig. 6). *Ophiostoma peniculi* is characterized by brush-like synnemata, which are absent in *O. hongxingense*, *O. subelongati* (see below), and *O. brunneolum* (Linnakoski et al. 2016).

*Additional specimens examined*: **China**: *Heilongjiang province*, Zhanhe, from *Ips subelongatus* infesting *Larix gmelinii*, Sept. 2017, *Q. Lu* (cultures CXY 2014 = CFCC 52688; CXY 1904); Jiamusi, from *Ips subelongatus* infesting *Larix olgensis*, Aug. 2011, *Q. Lu* (culture CXY 1920).

***Ophiostoma pseudobicolor*** Z. Wang & Q. Lu, **sp. nov.**

MycoBank MB 830615.

(Fig. 17)

**Fig. 17:**
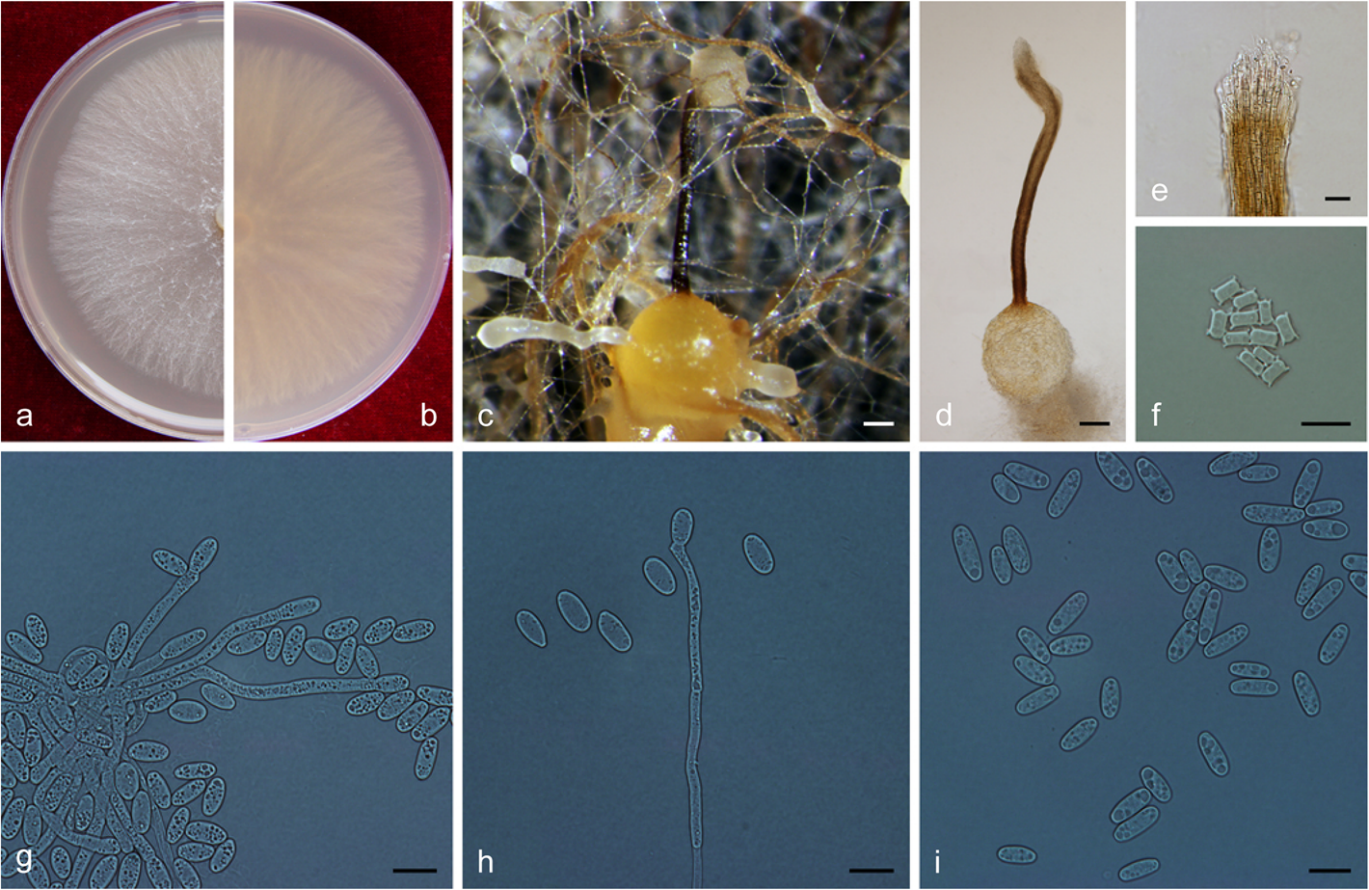
Morphological characteristics of *Ophiostoma pseudobicolor* sp. nov. (CFCC 52683, Taxon 8). **a–b**. Five-day-old cultures on 2% MEA; **c–d**. Perithecium. **e**. Apex of perithecium. **f**. Ascospores. **g–i**. Hyalorhinocladiella-like asexual morph: conidiogenous cells and conidia. Scale bars: c–d = 100 μm; e = 20 μm; f–i = 10 μm

*Etymology*: The epithet *pseudobicolor* (Latin) refers to the morphological resemblance and phylogenetic affinities with *O. bicolor*.

*Diagnosis*: *Ophiostoma pseudobicolor* is the closest phylogenetic relative to *O. bicolor*. Morphologically, these two species differ by the size of their perithecia, with globose bases ranging from (308–) 350–480 (− 536) μm in *O. pseudobicolor* vs. 175–350 (− 365) μm in *O. bicolor* (Upadhyay 1981). The necks of *O. pseudobicolor* are more robust than those of *O. bicolor*, especially at the apex [viz. (43–) 49–68 (− 77) μm vs. 15–42 (− 50) μm].

*Type*: **China**: *Inner Mongolia Autonomous Region*, Genhe, from *Ips subelongatus* infesting *Larix gmelinii*, Sept. 2017, *Q. Lu* (CXY 2009 – holotype, CFCC 52683 – ex-type culture).

*Description*: *Sexual morph* perithecial. *Perithecia* developing on 2% MEA at 25 °C after 15 d from superficial or embedded mycelium, superficial or partly embedded in the agar medium, with the base globose, subhyaline to yellow orange, (309–) 350–480 (− 536) μm diam., extending into a filiform neck; bases globose, prolonged by a cylindrical, straight or slightly curved, occasionally twinning neck, black (663–) 754–1100 (1358) μm long, (33–) 55–77 (− 79) μm wide at the base down to (43–) 49–68 (− 77) μm wide at the apex, composed of densely packed septate hyphae with a parallel orientation. *Ostiolar hyphae* absent. *Ascospores* hyaline, elliptical to oblong with obtuse ends in side and face view, circular in pole view, surrounded by a thick, hyaline gelatinous sheath appearing ossiform in side and face views, quadrangular with flanged corners in the end view, aseptate, (5.5–) 6–6.5 (− 7) × (3–) 3.5–4 (− 4.5) μm, included sheath.

*Asexual morph*: hyalorhinocladiella-like.

*Hyalorhinocladiella-like morph*: *conidiogenous cells* arising directly from the hyphae, (14–) 21.5–43.5 (− 60.5) × (2–) 2.5–4 (− 4.5) μm. *Conidia* hyaline, smooth, cylindrical, aseptate, (8.5–) 10–12 (− 13) × (4–) 4.5–5.5 (− 6) μm.

*Cultures*: *Colonies* on 2% MEA at 25 °C, fast growing, reaching 80 mm diam. in 5 d, effuse, cottony, hyaline to white at first, becoming white gray or gray brown; hyphae submerged in agar with many aerial mycelium. Optimal temperature for growth at 30 °C, no growth observed at 5 °C or 40 °C.

*Ecology*: Isolated from *Ips subelongatus* infesting dying *Larix gmelinii*, *L. olgensis* and *L. principis-rupprechtii*.

Habitat: *L. gmelinii*, *L. olgensis* or *L. principis-rupprechtii* pure plantation.

*Distribution*: Currently known from the Inner Mongolia Autonomous Region and Heilongjiang province, China.

*Notes*: *Ophiostoma pseudobicolor* is characterized by perithecium with a light-colored base extending into a dark neck. Similarly colored perithecia have been identified in *O. bicolor* (Upadhyay 1981), which also is the closest phylogenetic relative to *O. pseudobicolor* (Figs. 7, 8). However, DNA sequences of ITS and βT (Figs. 7, 8) clearly showed that both species represent two distinct clades.

*Additional specimens examined*: **China**: *Inner Mongolia Autonomous Region*, Genhe, from *Ips subelongatus* infesting *Larix gmelinii*, Sept. 2017, *Q. Lu* (cultures CXY 2010; CFCC 52684); Chifeng, from *Ips subelongatus* infesting *Larix principis-rupprechtii*, Aug. 2011, *Q. Lu* (cultures CFCC 52685; CXY 2011 = MUCL 55168; 1910); *Heilongjiang*, Mohe, from *Ips subelongatus* infesting *Larix principis-rupprechtii*, May 2012, *Q. Lu* (cultures CFCC 52686; CXY 2012 = MUCL 55174); Tahe, from *Ips subelongatus* infesting *Larix gmelinii*, May 2012, *Q. Lu* (culture CXY 1911 = MUCL 55170); Jiamusi, from *Ips subelongatus* infesting *Larix olgensis*, Aug. 2011, Q. Lu (culture CXY 1925; CXY1926).

***Ophiostoma subelongati*** Z. Wang & Q. Lu, **sp. nov.**

MycoBank MB 830616.

(Fig. 18)

**Fig. 18:**
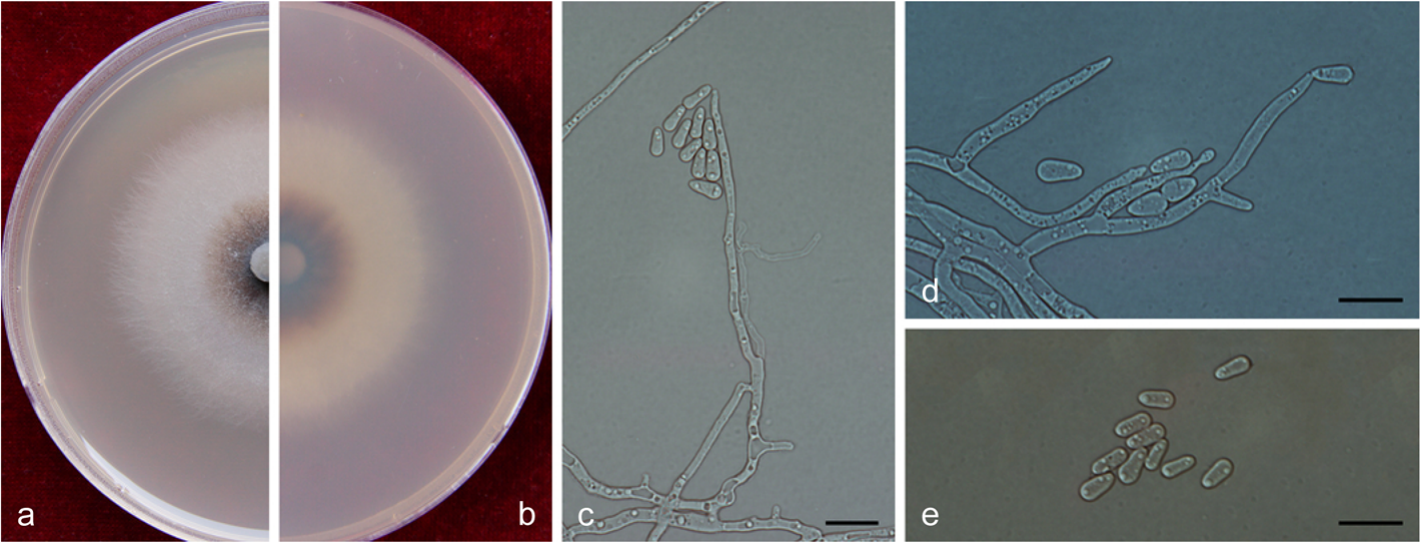
Morphological characteristics of *Ophiostoma subelongati* sp. nov. (CFCC 52693, Taxon 10). **a–b.** Five-day-old cultures on 2% MEA; **c–e.** Hyalorhinocladiella-like asexual morph: conidiogenous cells and conidia. Scale bars: c–e = 10 μm

*Etymology*: The epithet *subelongati* (Latin) refers to the vector (*Ips subelongatus*) from which this fungus was isolated.

*Diagnosis*: *Ophiostoma subelongati* colonies gradually turned brownish grey from the centre, but the *O. hongxingense* colonies centre turned dark olivaceous.

*Type*: **China**: *Heilongjiang province*, Hongxing, from *Ips subelongatus* infesting *Larix gmelinii*. July 2017, *Q. Lu* (CXY 2019 – holotype, CFCC 52693 – ex-type culture).

*Description*: *Sexual morph* not observed.

*Asexual morph*: hyalorhinocladiella-like.

*Hyalorhinocladiella-like morph*: *conidiogenous cells* arising directly from superficial hyphae, (11.5–) 12.5–27 (− 28) × 2–3 μm. *Conidia* hyaline, smooth, elliptical, aseptate, (4.5–) 5.5–7 (− 8.5) × 2.5–3.5 (− 4) μm.

*Cultures*: *Colonies* on 2% MEA at 25 °C reaching 61 mm diam. in 5 d, initially hyaline, the colonies edge thins radially, then from the centre of the colonies to the periphery it becomes brownish grey and develops superficial mycelium on the agar. Optimal temperature for growth is 30 °C; no growth observed at 5 °C or 40 °C.

*Ecology*: Isolated from *Ips subelongatus* infesting dying *Larix gmelinii* and *L. olgensis*.

Habitat: *L. gmelinii* or *L. olgensis* pure plantation.

*Distribution*: Currently known from the Inner Mongolia Autonomous Region and Heilongjiang province, China.

*Notes*: *Ophiostoma subelongati* forms a distinct clade within the *O. clavatum* complex (Linnakoski et al. 2016), in which it is closely related to *O. hongxingense*, *O. peniculi*, *O. macroclavatum*, *O. pseudocatenulatum*, and *O. brunneolum* (Fig. 6). These species share a similar hyalorhinocladiella-like state. *Ophiostoma peniculi*, *O. macroclavatum*, and *O. pseudocatenulatum* can be distinguished from *O. subelongati* and *O. hongxingense* by the presence of synnemata, which is absent from the latter two. The optimal growth temperature of *O. peniculi*, *O. subelongati*, and *O. hongxingense* is 30 °C, while that of *O. macroclavatum* and *O. brunneolum* is 25 °C. For *O. subelongati* and *O. hongxingense*, no growth observed at 5 °C and 40 °C, but *O. pseudocatenulatum* can still grow at 5 °C. In terms of colonies characteristics, *O. peniculi*, *O. subelongati*, and *O. hongxingense* grow faster than the above three species on 2% MEA at 25 °C.

*Additional specimens examined*: **China**: *Heilongjiang province*, Hongxing, from *Ips subelongatus* infesting *Larix gmelinii*, July 2017, *Q. Lu* (culture CXY 2020 = CFCC 52694; CXY 1921; CXY 1922); Jiamusi, from *Ips subelongatus* infesting *Larix olgensis*, Aug. 2011, *Q. Lu* (culture CXY 1923).

***Ophiostoma xinganense*** Z. Wang & Q. Lu, **sp. nov.**

MycoBank MB 830617.

(Fig. 19)

**Fig. 19:**
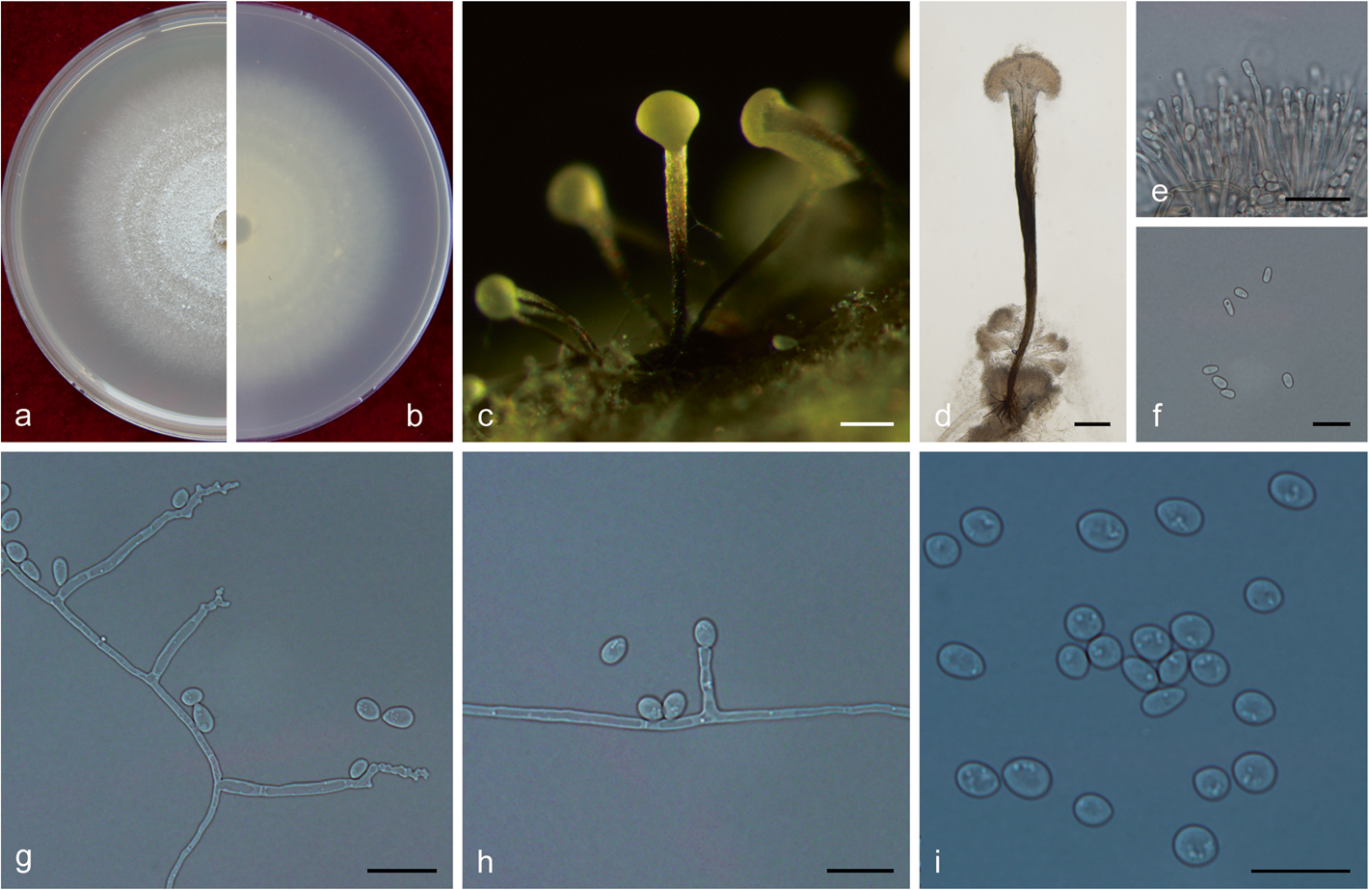
Morphological characteristics of *Ophiostoma xinganense* sp. nov. (CFCC 52679, Taxon 11). **a–b.** Ten-day-old culture on 2% MEA; **c–d.** Pesotum-like asexual morph; **e–f.** Conidiogenous cells of pesotum-like asexual morph and conidia; **g.** Sporothrix-like asexual morph: conidiogenous cells and conidia. **h–i.** Hyalorhinocladiella-like asexual morph: conidiogenous cells and conidia. Scale bars: c = 100 μm; d = 50 μm; e–i = 10 μm

*Etymology*: The epithet *xinganense* (Latin) refers to the Xing’an mountains from where this taxon was first isolated.

*Diagnosis*: *Ophiostoma xinganense* is closely related to *O. rufum*. *Ophiostoma xinganense* develops three synanamorphs, pesotum-like, sporothrix-like and hyalorhinocladiella-like asexual states, but *O. rufum* has pesotum-like and sporothrix-like states and lacks hyalorhinocladiella-like state (Jankowiak et al. 2019). Both sporothrix-like and pesotum-like asexual states have been observed in both species, but their conidia are different in shape and size. Conidia of sporothrix-like asexual state is ovate to oblong shape with 4–5.5 (− 7) × (2–) 2.5–4 (− 4.5) μm in *O. xinganense* vs. clavate or fusiform (primary conidia) with (7.2–) 8.9–12.4 (− 15.2) × (2–) 2.5–3.1 (− 3.4) μm in *O. rufum*, showing the former much smaller and rounder compared to the latter. Conidia of pesotum-like asexual state is ovate to oblong shape with 3.5–4.5 (− 5) × 2–2.5 μm in *O. xinganense* vs. oblong to curved shape with (2.5–) 3.3–4.5 (− 6.2) × (1.2–) 1.4–1.7 (− 2.2) μm in *O. rufum*, showing the former wider and rounder than the latter.

*Type*: **China**: *Inner Mongolia Autonomous Region*, Genhe, from *Ips subelongatus* infesting *Larix gmelinii*, Sept. 2017, *Q. Lu* (CXY 2005 – holotype, CFCC 52679 – ex-type culture).

*Description*: *Sexual morph* not observed.

*Asexual morphs*: pesotum-like, sporothrix-like and hyalorhinocladiella-like.

*Pesotum-like morph*: *synnemata* solitary or in groups, base black, (16.5–) 29.5–80 (− 114.5) μm wide, (446–) 483–768 (− 953) μm tall including the conidiogenous apparatus. *Conidiogenous cells* (11–) 13–23.5 (− 29.5) × (1–) 1.5–2 μm. *Conidia* hyaline, smooth, ovate to oblong, aseptate, 3.5–4.5 (− 5) × 2–2.5 μm. *Sporothrix-like morph*: *conidiogenous cells* arising directly from hyphae, (15.5–) 21.5–49.5 (− 79) × 1.5–2 (− 2.5) μm. *Conidia* hyaline, smooth, ovate to oblong, aseptate, 4–5.5 (− 7) × (2–) 2.5–4 (− 4.5) μm. *Hyalorhinocladiella-like morph*: *conidiogenous cells* arising directly from hyphae, (9.5–) 11–18.5 (− 23) × 1.5–2 (− 2.5) μm. *Conidia* hyaline, smooth, ovate to oblong, aseptate, (4–) 4.5–5 (− 5.5) × 3–4 μm.

*Cultures*: *Colonies* on 2% MEA at 25 °C reaching 75 mm diam. in 10 d, initially whitish gray, the colonies edge thinning radially; hyphae mostly superficial, sparsely aerial, synnemata developing abundantly in the colonies centre. Optimal temperature for growth at 25 °C, no growth observed at 5 °C and 40 °C.

*Ecology*: Isolated from *Ips subelongatus* infesting dying *Larix gmelinii* and stock log.

Habitat: *L. gmelinii* pure plantation.

*Distribution*: Currently only known from the Inner Mongolia Autonomous Region, China.

*Notes*: *Ophiostoma xinganense* is closely related to *O. rufum* and *O. brunneum* (Hausner et al. 2003, Jankowiak et al. 2019) (Fig. 3). *Ophiostoma xinganense* and *O. rufum* can be distinguished from *O. brunneum* by the presence of a pesotum-like asexual state, which is absent in the latter. In terms of colony characteristics, *O. xinganense* colonies are whitish gray with edge thinning radially and exhibits a clear concentric pattern of cream-colored rings, but *O. rufum* colonies were brownish orange to a rust brown with margin smooth and without concentric rings. Furthermore, at their optimal growth temperature (25 °C), physiologically *O. xinganense* shows a radial growth over three times faster than that reported in *O. rufum* under the same conditions on 2% MEA (7.5 mm/d vs. 2.2 mm/d, Jankowiak et al. 2019). In addition, *O. xinganense* can grow at 30 and 35 °C, but *O. rufum* can not at both temperatures.

*Additional specimens examined*: **China**: *Inner Mongolia Autonomous Region*, Genhe, from *Ips subelongatus* infesting *Larix gmelinii*, Sept. 2017, *Q. Lu* (cultures CXY 2006 = CFCC 52680; CXY 1901; CXY 1902; CXY 1903).

## DISCUSSION

In this study, 496 strains of ophiostomatoid fungi were obtained from adults and galleries of *Ips subelongatus* infesting *Larix gmelinii*, *L. olgensis*, *L. principis-rupprechtii*, and *Pinus sylvestris* var. *mongolica* in northeastern China. A combination of morphological and multi-locus phylogenetic approaches allowed identification of high diversity of ophiostomatoid fungi, encompassing 14 species belonging to four genera. They included eight previously undescribed *Ophiostoma* species, viz. *O. genhense*, *O. hongxingense*, *O. lotiforme*, *O. multisynnematum*, *O. peniculi*, *O. pseudobicolor*, *O. subelongati*, and *O. xinganense*. Two strains remain of uncertain status, and are *hitherto* referred to as *Ceratocystiopsis* cf. *pallidobrunnea*. Five known species also were recorded viz. *Ophiostoma minus*, *O. olgensis*, *O. rufum*, *Leptographium zhangii*, and *Endoconidiophora fujiensis.*

The dominant species were *O. peniculi*, *O. subelongati*, and *O. hongxingense* of the *O. clavatum* complex, and *Ophiostoma pseudobicolor* in the *O. ips* complex representing 23.8, 20.8, 17.7, and 14.5% of the isolates, respectively (Table 2). The fact that former three dominant species were not isolated from *Larix principis-rupprechtii* might be because *L. principis-rupprechtii* was less distributed in northeast China and there was only one sampling site for it. *Ophiostoma pseudobicolor* was the only species commonly encountered from three different larches (Table 2). Four species from *O. piceae* complex, *O. genhense*, *O. multisynnematum*, *O. rufum*, and *O. xinganense* were isolated only from *L. gmelinii* (Inner Mongolia) (Table 2). *Ophiostoma minus* was isolated from *L. gmelinii* and *Pinus sylvestris* var. *mongolica* (Inner Mongolia), and there were two unique species (*O. lotiforme* and *C.* cf. *pallidobrunnea*) isolated only from *P. sylvestris* var. *mongolica* (Inner Mongolia) (Table 2). *Leptographium zhangii* was previously reported to have been isolated only in Heilongjiang (*L. gmelinii*), but we also isolated it in Inner Mongolia (*L. gmelinii*). Meng et al. (2015) first determined that *Endoconidiophora fujiensis* extensively existed in three allopatric larch forests in northeast China, and we also isolated it from *L. gmelinii* in Heilongjiang (Table 2).

**Table 2:** Strains of ophiostomatoid fungi associated with *Ips subelongatus* in northeast China

Genus	Species	Distribution	Host	Numbers of isolates		Total	Total percentage
				Beetles	Galleries		
*Ophiostomatales*							
*Ophiostoma* s.l.	*O. genhense* sp. nov. (Taxon 1)	Inner Mongolia	*Larix gmelinii*	0	2	2	0.4
	*O. hongxingense* sp. nov. (Taxon 2)	Inner Mongolia and Heilongjiang	*L. gmelinii* and *L. olgensis*	71	17	88	17.7
	*O. lotiforme* sp. nov. (Taxon 3)	Inner Mongolia	*Pinus sylvestris* var. *mongolica*	0	2	2	0.4
	*O. minus* (Taxon 4)	Inner Mongolia	*L. gmelinii* and *P. sylvestris var. mongolica*	0	4	4	0.8
	*O. multisynnematum* sp. nov. (Taxon 5)	Inner Mongolia	*L. gmelinii*	0	9	9	1.8
	*O. olgensis* (Taxon 6)	Inner Mongolia and Heilongjiang	*L. gmelinii* and *L. olgensis*	21	17	38	7.7
	*O. peniculi* sp. nov. (Taxon 7)	Inner Mongolia and Heilongjiang	*L. gmelinii* and *L. olgensis*	39	79	118	23.8
	*O. pseudobicolor* sp. nov. (Taxon 8)	Inner Mongolia and Heilongjiang	*L. gmelinii*, *L. olgensis* and *L. principis-rupprechtii*	30	42	72	14.5
	*O. rufum* (Taxon 9)	Inner Mongolia	*L. gmelinii*	0	7	7	1.4
	*O. subelongati* sp. nov. (Taxon 10)	Inner Mongolia and Heilongjiang	*L. gmelinii* and *L. olgensis*	83	20	103	20.8
	*O. xinganense* sp. nov. (Taxon 11)	Inner Mongolia	*L. gmelinii*	0	17	17	3.4
*Ceratocystiopsis*	*C.* cf. *pallidobrunnea* (Taxon 12)	Inner Mongolia	*P. sylvestris* var. *mongolica*	0	2	2	0.4
*Leptographium* s.l.	*L. zhangii* (Taxon 13)	Inner Mongolia and Heilongjiang	*L. gmelinii*	13	3	16	3.2
*Microascales*							
*Endoconidiophora*	*E. fujiensis* (Taxon 14)	Heilongjiang	*L. gmelinii*	9	9	18	3.6
Total				266	230	496	100.0

In China, seven additional species of the *O. clavatum* complex have been recently described (Yin et al. 2016; Chang et al. 2017, 2019). Two of them are *Ophiostoma shangrilae* and *O. poligraphi*, which have been described based on isolates found in association with three bark beetles (viz. *Ips shangrila* and *Dendroctonus micans* infesting *Picea purpurea*; *Polygraphus poligraphus* and *D. micans* infesting *P. crassifolia*) from Qinghai province. The other four are *Ophiostoma jiamusiensis*, *O. songshui*, *O. ainoae*, and *O. brunneolum*, which have been described based on strains isolated from *I. typographus* infesting spruces in northeastern China (Yin et al. 2016; Chang et al. 2019). *Ophiostoma brevipilosi* was originally described from strains isolated from *Tomicus brevipilosus* infesting *Pinus kesiya* in Yunnan province (Chang et al. 2017). *Ophiostoma hongxingense*, *O. peniculi*, and *O*. *subelongati* are currently known from larch in two northeastern provinces in this study.

*Ophiostoma rufum* (Jankowiak et al. 2019), and three of the new species described here belong to the *O. piceae* complex (Harrington et al. 2001), which is mainly characterized by a synnematous, pesotum-like and sporothrix-like asexual state. Jankowiak et al. (2019) described *O. rufum* with a brownish orange to a rust brown colonies and a sporothrix-like asexual state. Our strains, however, differ in having whitish gray colonies and a hyalorhinocladiella-like asexual state (Additional file 10: Fig. S9). Whether these deviating characters are caused by intraspecific variation or different culture conditions remains unclear and needs to be further studied. To date, 10 species in the *O. piceae* complex have been recorded in China (Lu et al. 2009; Paciura et al. 2010a; Yin et al. 2016; Chang et al. 2017, 2019). Four species, *O. nitidum*, *O. micans*, *O. qinghaiense*, and *O. typographi*, have been described from China (Qinghai and Heilongjiang provinces) from spruces infested by *I. nitidus*, *I. typographus*, *D. micans*, and *Po. poligraphus* (Yin et al. 2016, Chang et al. 2019). Previously, *O. piceae* and *O. setosum* were reported to associated with *Larix*, *Pinus*, and *Tsuga* in Jilin and Yunnan provinces (Lu et al. 2009, Paciura et al. 2010a, Chang et al. 2017). The four new species here described were all isolated from *I subelongatus* infesting *L. gmelinii* in Inner Mongolia.

*Ophiostoma pseudobicolor* forms part of the *O. ips* complex (De Beer et al. 2013, De Beer & Wingfield 2013), in which it is related to *O. bicolor*, a species associated with various bark beetles in China (Chang et al. 2019), Japan (Yamaoka et al. 1997), and Europe (Upadhyay 1981, Linnakoski et al. 2010). These two species can be distinguished from each other by their genetic divergences, as evidenced by the phylogenetic analyses, but also by morphological data, such as the size of the ascocarps. Furthermore, their association with bark beetles and hosts affinities are differential too.

*Ophiostoma lotiforme* resided in a species complex previously reported as group A (Chang et al. 2017), together with *O. saponiodorum*, *O. pallidulum*, *O. acarorum*, and *O. massoniana* (Linnakoski et al. 2010, Wang et al. 2018). In our study, *O. lotiforme* was isolated from a single location (Inner Mongolia) from *Pinus sylvestris* var. *mongolica.*

Two known species from the *O. minus* complex (Gorton et al. 2004), *O. olgensis* and *O. minus*, were also recorded in our study. *Ophiostoma olgensis* was first described from the northeastern China, associated with *I. subelongatus* (Wang et al. 2016); it was again observed, and the species might be common in these northeastern China larch ecosystems. *Ophiostoma minus* has a wide distribution in northern hemisphere pine forests (Gorton & Webber 2000, Lu et al. 2009, Wang et al. 2019).

In a phylogenetic perspective, the *O. minus* lineage was subdivided into two clades, viz. a North American and an Eurasian clades, which are considered as two allopatric populations, each with a differential autecology as far as their host is concerned. The North American population is associated with *Dendroctonus* spp., whereas the Eurasian population is associated with various pine-infesting beetles (Gorton & Webber 2000, Lu et al. 2009). In a previous study of ophiostomatoid species associated with *Tomicus* species infesting pines in Yunnan, southwestern China, strains of *O. minus* formed a distinct, third clade, which was interpreted as a third allopatric population (Wang et al. 2019). In the current study, our strains of *O. minus* clustered together with the Eurasian population (Figs. 4, 5) and not with the Yunnan population (Wang et al. 2019). The origin, worldwide dispersion, and insect relationships range of these populations still require further studies.

Four *Leptographium* species have also been isolated from *Ips subelongatus* in northeast China to date (Paciura et al. 2010b, Liu et al. 2016). *Leptographium zhangii*, which was observed also in our study, has previously been collected from other parts of northeastern China (Liu et al. 2016), confirming its widespread occurrence in the region.

*Ips subelongatus* and *I. cembrae* have been long considered as a single species with a wide distribution range. Their fungal associates were also thought to be generally identical over the presumed beetle geographic distribution range (Wood & Bright 1992, Yamaoka et al. 1998, Stauffer et al. 2001). In particular, *Endoconidiophora laricicola*, a pioneer invader and the most virulent fungal associate, has also been considered as widespread fungus, following the distribution of the beetle (Yamaoka et al. 1998, Stauffer et al. 2001). However, accurate comparison of specimens of eight spined larch bark beetles from Europe and Asia showed two allopatric species, corresponding to *I. cembrae* and *I. subelongatus* (Stauffer et al. 2001). In parallel, Japanese strains of *E. laricicola* associated with *I. subelongatus* were shown by multigene phylogenetic inferences to also represent a distinct species, *E. fujiensis* (Marin et al. 2005). Another species in genus the *Endoconidiophora*, i.e.*, E. polonica*, is associated with subspecies and/or distinct geographic populations of *I. typographus* was also shown to represent two distinct populations, that may have coevolved with the two allopatric populations of their beetle vector, *I. typographus* (Stauffer & Lakatos 2000, Marin et al. 2009). Beetle and fungus speciation seemed to occur concomitantly with dispersal.

In China, Meng et al. (2015) reported that *E. fujiensis* is widely distributed in three larch forests in northeastern China, forming a stable association with *I. subelongatus* under such ecological conditions. In the present study, *E. fujiensis* was again collected from this area, supporting the previous observations.

The pathogenicity of the Chinese strains of *E. fujiensis* was tested by inoculation on mature, both native and introduced, larches in the field (Liu 2015). In that study, Chinese strains of *E. fujiensis* caused limited necrotic areas (approx. 5 cm in length over 2 months) in the three native larches, whereas it caused necrosis of more than 70 cm in length (over 2 months) in Japanese larch (*L. kaempferi*) (Liu 2015), results that are very similar to those of a previous report (Yamaoka et al. 1998). The Japanese larch was introduced and has been planted over very large areas of China, from northeastern to northwestern provinces (e.g., Gansu province) down to the southern provinces (e.g., Hubei) because of its rapid growth and stress resistance (China Flora Editorial Committee of Chinese Academy of Sciences 1978, Ma & Wang 1990, Zhu et al. 2015). Therefore, the introduction and extensive afforestation by Japanese larch in China needs careful consideration and reevaluation because of its high susceptibility to forest pathogens.

**Fig. 20:**
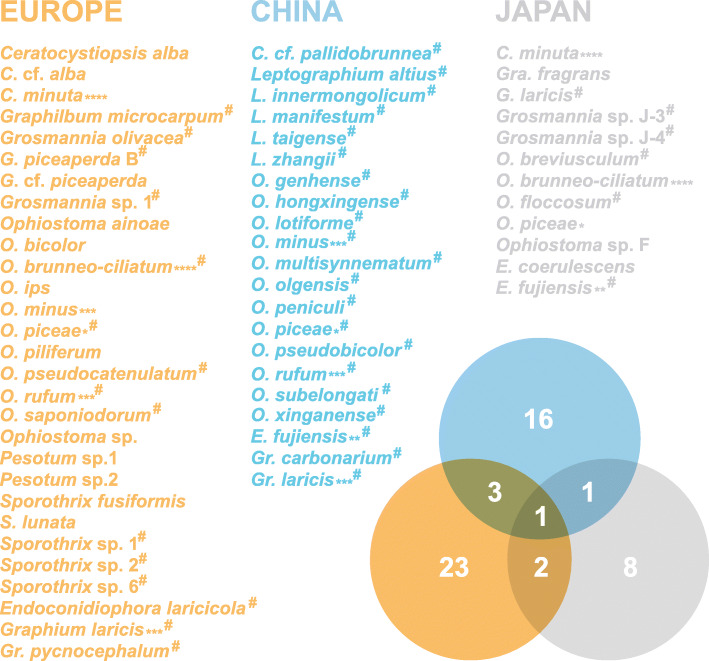
Venn diagram showing overlaps of the ophiostomatoid fungal communities associated with *Ips cembrae* in Europe and *I. subelongatus* in China and Japan * species common to all three regions; ** species common to both China and Japan; *** species common to both China and Europe; **** species common to both Europe and Japan; # species identity confirmed by molecular data.

The ophiostomatoid fungi associated with *I. cembrae* and *I. subelongatus* in Palaearctic larch forests have been investigated extensively and is well documented also in Europe and Japan (Additional file 1: Table S1 and references cited therein). Based on the data available, 54 species were identified as being associated with *I. cembrae* and *I. subelongatus* infesting larches. *Hitherto*, the highest species diversity was observed in European larch forests, followed by those in China and Japan, with 29, 21, and 12 species recorded locally, respectively (Fig. 20, Additional file 1: Table S1). However, this might still reflect incomplete survey in Eastern Asia, especially China. The direct comparison among the ophiostomatoid communities is difficult. Seven species have a known distribution range extending over two or three regions. Two species are shared by China and Japan, whereas four species are shared by China and Europe, and three by Europe and Japan (Fig. 20). The 47 other species are endemic to a single region. This high level of endemism might be explained by the endemism of both the beetle vector and larch species and by the wide geographical differences. A similar conclusion was drawn from comparisons among fungal assemblages associated with *I. typographus* (Chang et al. 2019).

*Ophiostoma piceae* is the only species shared among Europe, Japan, and China (Fig. 20). However, in Japan, the species associated with *I. subelongatus* were identified based solely on morphological characteristics, which is poorly informative in the *O. piceae* complex. This complex has recently been greatly enriched based on multilocus DNA sequence comparisons and further phylogenetic analyses (Linnakoski et al. 2010; Yin et al. 2016; Jankowiak et al. 2017; Chang et al. 2017, 2019). It seems clear that the identity of the Japanese *O. piceae* complex strains must be reevaluated with the aid of multiple gene sequences.

*Ophiostoma minus*, *O. rufum*, and *Graphium laricis* occur both in Europe and northeastern China (Pashenova et al. 1995, 2004; Kirisits et al. 2000; Stauffer et al. 2001; Jacobs et al. 2003; Kirisits 2004; Jankowiak et al. 2007; Linnakoski et al. 2010; Liu et al. 2016; Jankowiak et al. 2019). *Ophiostoma minus* seems to be undergoing population differentiation or a speciation process (Figs. 4, 5, Wang et al. 2019). *Ceratocystiopsis minuta* and *O. brunneo-ciliatum* were reported as present in both Europe and Japan (Aoshima 1965; Redfern et al. 1987; Redfern 1989; Yamaoka et al. 1998, 2009; Kirisits et al. 2000; Stauffer et al. 2001; Kirisits 2004; Jankowiak et al. 2007; Jankowiak et al. 2017; Yamaoka 2017); however, the reports of their existence in Japan also relied only on morphological identification, and would require molecular confirmation. This is particularly pertinent for *O. brunneo-ciliatum*, which is one of more frequently reported species associated with *I. subelongatus* in Japan (Aoshima 1965; Yamaoka et al. 1998, 2009; Yamaoka 2017).

*Endoconidiophora fujiensis* is the only species that is extensively associated with *I. subelongatus* in northeastern Asian larch forests. A concatenate phylogenetic analysis showed a genetic differentiation within this species, much higher than the intraspecific various of two sibling species, *E. polonica* and *E. laricicola* (Meng et al. 2015). These findings are consistent with the possible differentiation of the beetle vector *I. subelongatus* (Zhang et al. 2007, Song et al. 2011, Chen et al. 2016).

## CONCLUSIONS

The results of this study indicate a high diversity of ophiostomatoid species associated with *I. subelongatus* infestations of larch and pine forests in northeastern China. Fourteen species were identified, of which eight *Ophiostoma* species were new to science. The dominant species were *O. peniculi*, *O. hongxingense*, and *O. subelongati* in the *O. clavatum* complex and *O. pseudobicolor* in the *O. ips* complex. The comparisons among ophiostomatoid communities associated with *I. subelongatus* in China and Japan, and with *I. cembrae* in Europe showed distinct assemblage patterns. The difference between Asian and European communities might be reasonable due to huge geographical distance and quite different environments, but was unexpected for the difference between northeastern Chinese and Japanese communities. However, the conclusion still need to be confirmed though molecular identification on all species compositions. As a pioneer invader, *E. fujiensis* caused noticeable necrosis to Japanese larch (*L. kaempferi*) but seemed weakly virulent to the local larches (Liu 2015). Therefore, the introduction and extensive afforestation of Japanese larch in China needs careful consideration and reevaluation because of its high susceptibility to this forest pathogen.

## Supplementary information

**Additional file 1: Table S1.** Comparison of ophiostomatoid associates of *Ips cembrae* or *I. subelongatus* in Europe, China, and Japan

**Additional file 2: Figure S1.** ML tree of *O. piceae* complex generated from the βT sequence data. Sequences generated from this study are printed in bold. Bold branches indicate posterior probability values ≥0.9. Bootstrap values of ML/MP ≥ 70% are recorded at the nodes. T = ex-type isolates

**Additional file 3: Figure S2.** ML tree of *O. piceae* complex generated from the EF-1α sequence data. Sequences generated from this study are printed in bold. Bold branches indicate posterior probability values ≥0.9. Bootstrap values of ML/MP ≥ 70% are recorded at the nodes. T = ex-type isolates

**Additional file 4: Figure S3.** ML tree of *O. piceae* complex generated from the CAL sequence data. Sequences generated from this study are printed in bold. Bold branches indicate posterior probability values ≥0.9. Bootstrap values of ML/MP ≥ 70% are recorded at the nodes. T = ex-type isolates

**Additional file 5: Figure S4.** ML tree of *O. clavatum* complex generated from the βT sequence data. Sequences generated from this study are printed in bold. Bold branches indicate posterior probability values ≥0.9. Bootstrap values of ML/MP ≥ 70% are recorded at the nodes. T = ex-type isolates

**Additional file 6: Figure S5.** ML tree of *O. clavatum* complex generated from the EF-1α sequence data. Sequences generated from this study are printed in bold. Bold branches indicate posterior probability values ≥0.9. Bootstrap values of ML/MP ≥ 70% are recorded at the nodes. T = ex-type isolates

**Additional file 7: Figure S6.** ML tree of *O. clavatum* complex generated from the CAL sequence data. Sequences generated from this study are printed in bold. Bold branches indicate posterior probability values ≥0.9. Bootstrap values of ML/MP ≥ 70% are recorded at the nodes. T = ex-type isolates

**Additional file 8: Figure S7.** ML tree of *L. zhangii* generated from the EF-1α sequence data. Sequences generated from this study are printed in bold. Bold branches indicate posterior probability values ≥0.9. Bootstrap values of ML/MP ≥ 70% are recorded at the nodes. T = ex-type isolates

**Additional file 9: Figure S8.** ML tree of *Endoconidiophora* generated from the 60S sequence data. Sequences generated from this study are printed in bold. Bold branches indicate posterior probability values ≥0.9. Bootstrap values of ML/MP ≥ 70% are recorded at the nodes. T = ex-type isolates

**Additional file 10: Figure S9.** Morphological characteristics of *Ophiostoma rufum* (CFCC 52681 Taxon 9). **a–b.** Ten-day-old cultures on 2% MEA; **c–d.** Pesotum-like asexual morph; **e–f.** Conidiogenous cells of pesotum-like asexual morph and conidia; **g–i.** Hyalorhinocladiella-like asexual morph: conidiogenous cells and conidia. Scale bars: c = 50 μm; d = 20 μm; e–i = 10 μm

## Data Availability

Not applicable.

## Abbreviations

60S: the partial 60S ribosomal protein RPL10 gene
BCCM/MUCL: the Mycothèque of the Université Catholique de Louvain, Belgium
BI: Bayesian inference
βT: the β-tubulin gene region
*C*: *Ceratocystiopsis*
CAL: the calmodulin gene region
CFCC: the China Forestry Culture Collection Centre
CXY: the culture collection of the Chinese Academy of Forestry
*D*: *Dendroctonus*
*E*: *Endoconidiophora*
EF-1α: the transcription elongation factor-1α gene region
*G*: *Grosmannia*
*Gr*: *Graphium*
*Gra*: *Graphilbum*
*I*: *Ips*
ITS: Internal transcribed spacer regions 1 and 2 of the nuclear ribosomal DNA operon, including the 5.8S region
*L*: *Larix*
*L*: *Leptographium*
LSU: the nuclear ribosomal large subunit region
MEA: Malt extract agar
ML: Maximum likelihood
MP: Maximum parsimony
*O*: *Ophiostoma*
*P*: *Picea*
*Po*: *Polygraphus*
s. l: Sensu lato
