# Correction to: Ophiostomatoid fungi associated with Ips subelongatus, including eight new species from northeastern China

**DOI:** 10.1186/s43008-020-00032-0

**Published:** 2020-04-20

**Authors:** Zheng Wang, Ya Liu, Huimin Wang, Xianjing Meng, Xuewei Liu, Cony Decock, Xingyao Zhang, Quan Lu

**Affiliations:** 1 Key Laboratory of Forest Protection, National Forestry and Grassland Administration; Research Institute of Forest Ecology, Environment and Protection, Chinese Academy of Forestry, Beijing, 100091 China; 2 Wuqing Forestry Bureau, Tianjin, 301700 China; 3 Mycothèque de l’Université Catholique de Louvain (MUCL), Earth and Life Institute, Microbiology, B-1348 Louvain-la-Neuve, Belgium

**Correction to: IMA Fungus (2020) 11:3**

**https://doi.org/10.1186/s43008-019-0025-3**

Following publication of the original article (Wang et al. 2020), the authors reported that Table 1 contained some errors in the GenBank number of BT gene. The correct Table 1 is presented below.

**Table. 1:** Strains of ophiostomatoid fungi sequenced and used for morphological and phylogenetic analysis in this study

Taxon	Species^1^	Strain no.^2.3.4^	Host	Locality	GenBank number^5^			
					ITS/LSU/60S	βT	EF-1α	CAL
1	***Ophiostoma genhense***	CFCC 52675 (CXY 2001) T	*Larix gmelinii*	Genhe, Inner Mongolia	MK748199	MN896053	MN896074	MN896102
		CFCC 52676 (CXY 2002)	*L. gmelinii*	Genhe, Inner Mongolia	N/A	MN896052	MN896073	MN896101
2	***O. hongxingense***	CFCC 52695 (CXY 2021) T	*L. gmelinii*	Harbin, Heilongjiang	MK748194	MN896026	MN896068	MN896089
		CFCC 52696 (CXY 2022)	*L. gmelinii*	Harbin, Heilongjiang	N/A	MN896028	MN896067	MN896090
		CXY 1905	*L. gmelinii*	Harbin, Heilongjiang	N/A	MN896027	MN896066	MN896087
		CXY 1906	*L. gmelinii*	Harbin, Heilongjiang	N/A	MN896030	MN896070	MN896088
		CXY 1907	*L. gmelinii*	Harbin, Heilongjiang	N/A	MN896029	MN896069	MN896091
3	***O. lotiforme***	CFCC 52691 (CXY 2017 = MUCL 55165) T	*Pinus sylvestris* var. *mongolica*	Hailar, Inner Mongolia	MK748185	MN896036	N/A	N/A
		CFCC 52692 (CXY 2018)	*P. sylvestris* var. *mongolica*	Hailar, Inner Mongolia	MK748201	MN896037	N/A	N/A
4	*O. minus*	CFCC 52697 (CXY 2023)	*L. gmelinii*	Arongqi, Inner Mongolia	MK748202	MN896044	N/A	N/A
		CFCC 52698 (CXY 2024 = MUCL 55157)	*P. sylvestris* var. *mongolica*	Hailar, Inner Mongolia	MK748187	MN896045	N/A	N/A
5	***O. multisynnematum***	CFCC 52677 (CXY 2003) T	*L. gmelinii*	Genhe, Inner Mongolia	MK748196	MN896050	MN896071	MN896103
		CFCC 52678 (CXY 2004)	*L. gmelinii*	Genhe, Inner Mongolia	N/A	MN896051	MN896072	MN896104
6	*O. olgensis*	CFCC 52699 (CXY 2025)	*L. gmelinii*	Genhe, Inner Mongolia	MK748204	MN896048	N/A	N/A
		CFCC 52700 (CXY 2026)	*L. gmelinii*	Genhe, Inner Mongolia	MK748195	MN896049	N/A	N/A
		CXY 1908	*L. gmelinii*	Harbin, Heilongjiang	MK748203	MN896046	N/A	N/A
		CXY 1909	*L. gmelinii*	Yichun, Heilongjiang	MK748205	MN896047	N/A	N/A
7	***O. peniculi***	CFCC 52687 (CXY 2013) T	*L. gmelinii*	Genhe, Inner Mongolia	MK748198	MN896034	MN896063	MN896086
		CFCC 52688 (CXY 2014)	*L. gmelinii*	Genhe, Inner Mongolia	N/A	MN896033	MN896061	MN896084
		CXY 1904	*L. gmelinii*	Genhe, Inner Mongolia	N/A	MN896035	MN896062	MN896085
8	***O. pseudobicolor***	CFCC 52683 (CXY 2009) T	*L. gmelinii*	Genhe, Inner Mongolia	MK748188	MN896038	N/A	N/A
		CFCC 52684 (CXY 2010)	*L. gmelinii*	Genhe, Inner Mongolia	MK748190	MN896040	N/A	N/A
		CFCC 52685 (CXY 2011 = MUCL 55168)	*L. principis-rupprechtii*	Chifeng, Inner Mongolia	MK748191	MN896043	N/A	N/A
		CFCC 52686 (CXY 2012 = MUCL 55174)	*L. gmelinii*	Mohe, Heilongjiang	MK748193	MN896041	N/A	N/A
		CXY 1910	*L. principis-rupprechtii*	Chifeng, Inner Mongolia	MK748192	MN896039	N/A	N/A
		CXY 1911 (MUCL 55170)	*L. gmelinii*	Tahe, Heilongjiang	MK748189	MN896042	N/A	N/A
9	*O. rufum*	CFCC 52681 (CXY 2007)	*L. gmelinii*	Genhe, Inner Mongolia	MK748197	MN896058	MN896075	MN896095
		CFCC 52682 (CXY 2008)	*L. gmelinii*	Genhe, Inner Mongolia	N/A	MN896057	MN896076	MN896098
10	***O. subelongati***	CFCC 52693 (CXY 2019) T	*L. gmelinii*	Harbin, Heilongjiang	MK748200	MN896031	MN896064	MN896092
		CFCC 52694 (CXY 2020)	*L. gmelinii*	Harbin, Heilongjiang	N/A	MN896032	MN896065	MN896093
11	***O. xinganense***	CFCC 52679 (CXY 2005) T	*L. gmelinii*	Genhe, Inner Mongolia	MK748186	MN896055	MN896078	MN896097
		CFCC 52680 (CXY 2006)	*L. gmelinii*	Genhe, Inner Mongolia	N/A	MN896054	MN896079	MN896094
		CXY 1901	*L. gmelinii*	Genhe, Inner Mongolia	N/A	MN896056	MN896077	MN896096
		CXY 1902	*L. gmelinii*	Genhe, Inner Mongolia	N/A	MN896059	MN896080	MN896099
		CXY 1903	*L. gmelinii*	Genhe, Inner Mongolia	N/A	MN896060	MN896081	MN896100
12	*Ceratocystiopsis* cf. *pallidobrunnea*	CFCC 52689 (CXY 2015)	*P. sylvestris* var. *mongolica*	Hailar, Inner Mongolia	MN892641	N/A	N/A	N/A
		CFCC 52690 (CXY 2016)	*P. sylvestris* var. *mongolica*	Hailar, Inner Mongolia	MN892642	N/A	N/A	N/A
13	*Leptographium zhangii*	CFCC 52701 (CXY 2027)	*L. gmelinii*	Genhe, Inner Mongolia	N/A	N/A	MN896082	N/A
		CFCC 52702 (CXY 2028)	*L. gmelinii*	Genhe, Inner Mongolia	N/A	N/A	MN896083	N/A
14	*Endoconidiophora fujiensis*	CXY 1912	*L. gmelinii*	Yichun, Heilongjiang	MN896105	N/A	N/A	N/A
		CXY 1913	*L. gmelinii*	Yichun, Heilongjiang	MN896106	N/A	N/A	N/A

1. Species named in black bold are novel species in this study

2. *CFCC* China Forestry Culture Collection Center, Beijing, China

3. *CXY* the culture collection of the Chinese Academy of Forestry

4. T = ex-holotype isolate

5. *ITS* Internal transcribed spacer regions 1 and 2 of the nuclear ribosomal DNA operon, including the 5.8S region, *LSU* Large subunit of the nrDNA, *60S* partial 60S

ribosomal protein RPL10 gene, *βT* the β-tubulin gene region, *EF-1α* the transcription elongation factor-1α gene region, *CAL* the calmodulin gene region
