# Supplementary material for: Ophiostomatoid fungi associated with Ips subelongatus, including eight new species from northeastern China
Source: IMA Fungus. 2020 Jan 31;11:3. doi: 10.1186/s43008-019-0025-3 (PMC7325231; doi:10.1186/s43008-019-0025-3)
Supplement: Supplementary file 1 — Additional file 1: Table S1. Comparison of ophiostomatoid associates of Ips cembrae or I. subelongatus in Europe, China, and Japan [file 43008_2019_25_MOESM1_ESM.docx]

Supplementary Table. 1 Comparison of ophiostomatoid associates of *Ips cembrae* or *I. subelongatus* in Europe, China and Japan

| Fungal species | Genus | *Ips cembrae* |  | *I. subelongatus* | |
| --- | --- | --- | --- | --- | --- |
|  |  | Europe |  | China | Japan |
| ***Ophiostomatales*** |  |  |  |  |  |
| *Ceratocystiopsis alba* | *Ceratocystiopsis* spp. | Jankowiak *et al.* 2007 |  |  |  |
| *C.* cf. *alba* |  | Kirisits *et al.* 2000; Stauffer *et al.* 2001; Kirisits 2004; |  |  |  |
| *C. minuta* |  | Kirisits *et al.* 2000; Stauffer *et al.* 2001; Kirisits 2004; Jankowiak *et al.* 2007 |  |  | Yamaoka *et al.* 1998, 2009; Yamaoka 2017 |
| *C.* cf. *pallidobrunnea* |  |  |  | this study |  |
| *Graphilbum fragrans* | *Graphilbum* spp. |  |  |  | Masuya *et al.* 2009; Yamaoka 2017 |
| *Gra. microcarpum* |  | Jankowiak *et al.* 2017 |  |  |  |
| *Grosmannia laricis* | *Leptographium* spp. |  |  |  | Westhuizen *et al.* 1995; Yamaoka *et al.* 1998, 2009; Ando *et al.* 2016; Yamaoka 2017 |
| *G. olivacea* |  | Jankowiak *et al.* 2017 |  |  |  |
| *G. piceaperda* B |  | Jankowiak *et al.* 2017 |  |  |  |
| *G.* cf. *piceaperda* |  | Jankowiak *et al.* 2017 |  |  |  |
| *Grosmannia* sp. 1 |  | Jankowiak *et al.* 2017 |  |  |  |
| *Grosmannia* sp. J-3 |  |  |  |  | Yamaoka *et al.* 2009; Ando *et al.* 2016; Yamaoka 2017 |
| *Grosmannia* sp. J-4 |  |  |  |  | Yamaoka *et al.* 2009; Ando *et al.* 2016; Yamaoka 2017 |
| *Leptographium altius** |  |  |  | Paciura *et al*. 2010b |  |
| *L. innermongolicum* |  |  |  | Liu *et al.* 2016 |  |
| *L. manifestum* |  |  |  | Paciura *et al*. 2010b |  |
| *L. taigense* |  |  |  | Liu *et al.* 2016 |  |
| *L. zhangii* |  |  |  | Liu *et al.* 2016; this study |  |
| *Ophiostoma ainoae* | *Ophiostoma* spp. | Pashenova *et al.* 1995; Linnakoski *et al.* 2010 |  |  |  |
| *O. bicolor* |  | Pashenova *et al.* 1995, 2004; Kirisits *et al.* 2000; Stauffer *et al.* 2001; Kirisits 2004; Linnakoski *et al.* 2010 |  |  |  |
| *O. breviusculum* |  |  |  |  | Chung *et al.* 2006; Yamaoka *et al.* 2009 |
| *O. brunneo-ciliatum* |  | Redfern *et al.* 1987; Redfern 1989; Kirisits *et al.* 2000; Stauffer *et al.* 2001; Kirisits 2004; Jankowiak *et al.* 2007; Jankowiak *et al.* 2016 |  |  | Aoshima 1965; Yamaoka *et al.* 1998, 2009; Yamaoka 2017 |
| *O. floccosum* |  |  |  |  | Aoshima 1965; Chung *et al.* 2006; Yamaoka *et al.* 2009; Yamaoka 2017 |
| *O. genhense* |  |  |  | this study |  |
| *O. hongxingense* |  |  |  | this study |  |
| *O. ips* |  | Pashenova *et al.* 1995; Jankowiak *et al.* 2007; Linnakoski *et al.* 2010 |  |  |  |
| *O. lotiforme* |  |  |  | this study |  |
| *O. minus* |  | Pashenova *et al.* 1995, 2004; Linnakoski *et al.* 2010 |  | this study |  |
| *O. multisynnematum* |  |  |  | this study |  |
| *O. olgensis* |  |  |  | Wang *et al*. 2016; this study |  |
| *O. peniculi* |  |  |  | this study |  |
| *O. piceae* |  | Stauffer *et al.* 2001; Kirisits 2004; Jankowiak *et al.* 2007; Jankowiak *et al.* 2017 |  | Paciura *et al*. 2010a | Aoshima 1965; Yamaoka *et al.* 1998, 2009; Yamaoka 2017 |
| *O. piliferum* |  | Pashenova *et al.* 1995, 2004; Linnakoski *et al.* 2010 |  |  |  |
| *O. pseudobicolor* |  |  |  | this study |  |
| *O. pseudocatenulatum* |  | Jankowiak *et al.* 2017 |  |  |  |
| *O. rufum* |  | Jankowiak *et al.* 2019 |  | this study |  |
| *O. saponiodorum* |  | Jankowiak *et al.* 2017 |  |  |  |
| *O. subelongati* |  |  |  | this study |  |
| *O. xinganense* |  |  |  | this study |  |
| *Ophiostoma* sp. |  | Jankowiak *et al.* 2007 |  |  |  |
| *Ophiostoma* sp. F |  |  |  |  | Yamaoka *et al.* 2009; |
| *Pesotum* sp.1 | unknown | Jankowiak *et al.* 2007 |  |  |  |
| *Pesotum* sp.2 |  | Jankowiak *et al.* 2007 |  |  |  |
| *Sporothrix fusiformis* | *Sporothrix* spp. | Aghayeva *et al.* 2004; Kirisits 2004 |  |  |  |
| *S. lunata* |  | Aghayeva *et al.* 2004; Kirisits 2004 |  |  |  |
| *Sporothrix* sp. 1 |  | Jankowiak *et al.* 2017 |  |  |  |
| *Sporothrix* sp. 2 |  | Jankowiak *et al.* 2017 |  |  |  |
| *Sporothrix* sp. 6 |  | Jankowiak *et al.* 2017 |  |  |  |
| ***Microascales*** |  |  |  |  |  |
| *Endoconidiophora coerulescens* | *Endoconidiophora* spp. |  |  |  | Yamaoka *et al.* 2009; Yamaoka 2017 |
| *E. fujiensis* |  |  |  | this study | Yamaoka *et al.* 1998, 2009; Yamaoka 2017 |
| *E. laricicola* |  | Redfern *et al.* 1987; Redfern 1989; Stauffer *et al.* 2001; Kirisits 2004; Jankowiak *et al.* 2007 |  |  |  |
| *Graphium carbonarium* | *Graphium* spp. |  |  | Liu *et al.* 2016 |  |
| *Gr. laricis* |  | Kirisits *et al.* 2000; Stauffer *et al.* 2001; Jacobs *et al.* 2003; Kirisits 2004; Jankowiak *et al.* 2007 |  | Liu *et al.* 2016 |  |
| *Gr. pycnocephalum* |  | Jankowiak *et al.*2007 |  |  |  |
| Number of species reported |  | 29 |  | 21 | 12 |

*. This species was reported association with *I. cembrae* in northeast China in original paper. The beetle should be interpreted as *I. subelongatus* (Stauffer *et al.* 2001).
