# Supplementary figures and images for: Ophiostomatoid fungi associated with Ips subelongatus, including eight new species from northeastern China
Source: IMA Fungus. 2020 Jan 31;11:3. doi: 10.1186/s43008-019-0025-3 (PMC7325231; doi:10.1186/s43008-019-0025-3)

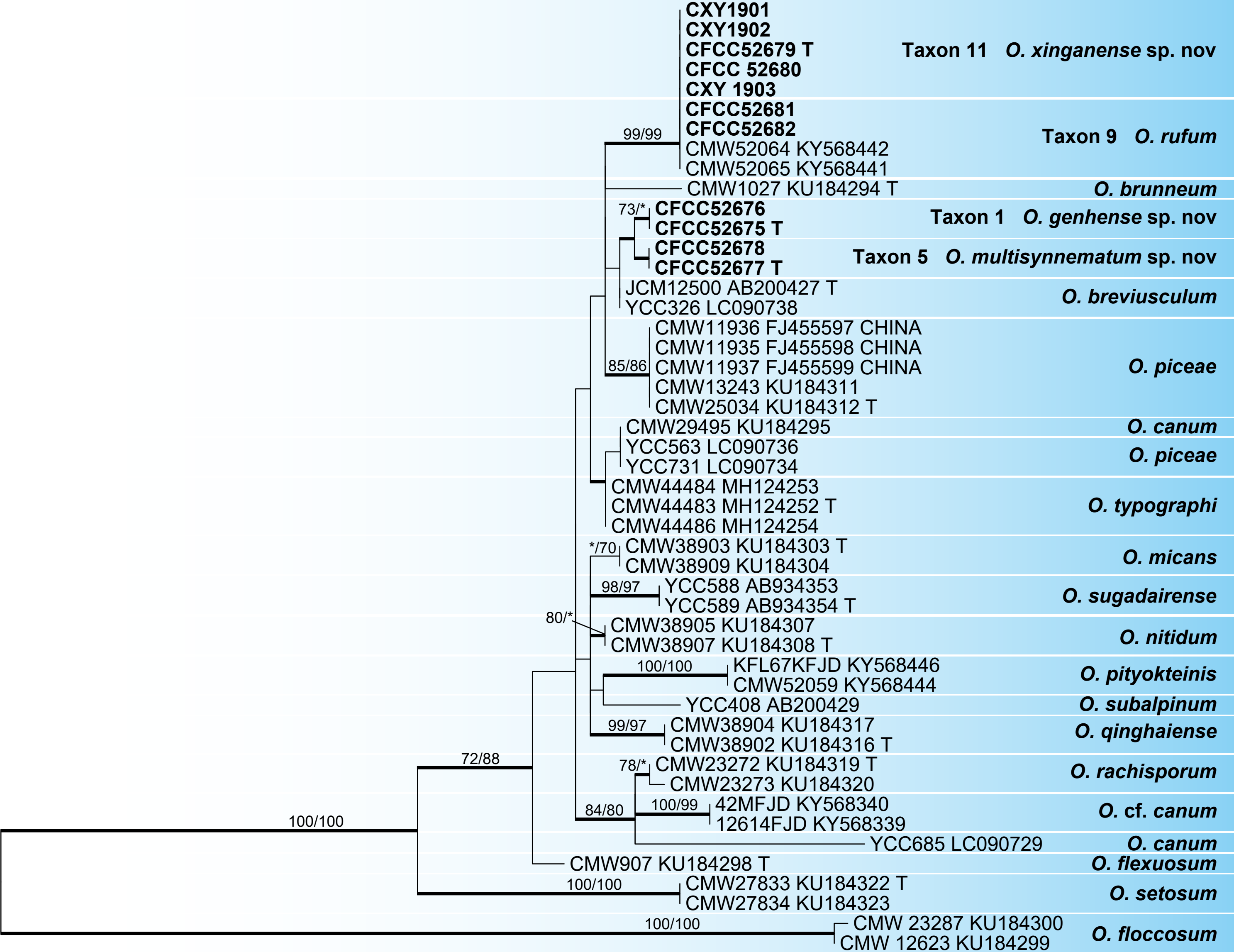

Supplement: Supplementary file 2 — Additional file 2: Figure S1. ML tree of O. piceae complex generated from the βT sequence data. Sequences generated from this study are printed in bold. Bold branches indicate posterior probability values ≥0.9. Bootstrap values of ML/MP ≥ 70% are recorded at the nodes. T = ex-type isolates [file 43008_2019_25_MOESM2_ESM.pdf]

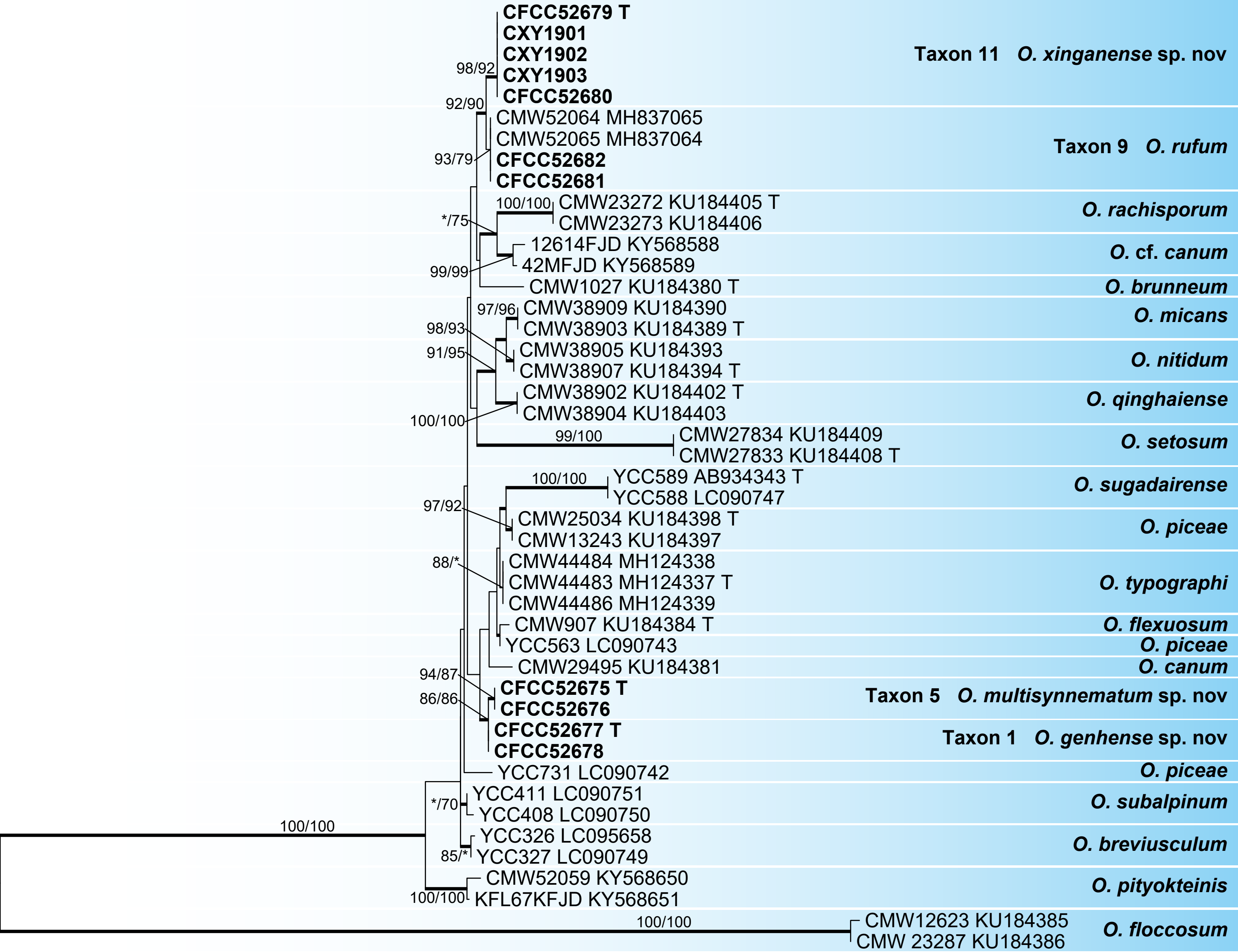

Supplement: Supplementary file 3 — Additional file 3: Figure S2. ML tree of O. piceae complex generated from the EF-1α sequence data. Sequences generated from this study are printed in bold. Bold branches indicate posterior probability values ≥0.9. Bootstrap values of ML/MP ≥ 70% are recorded at the nodes. T = ex-type isolates [file 43008_2019_25_MOESM3_ESM.pdf]

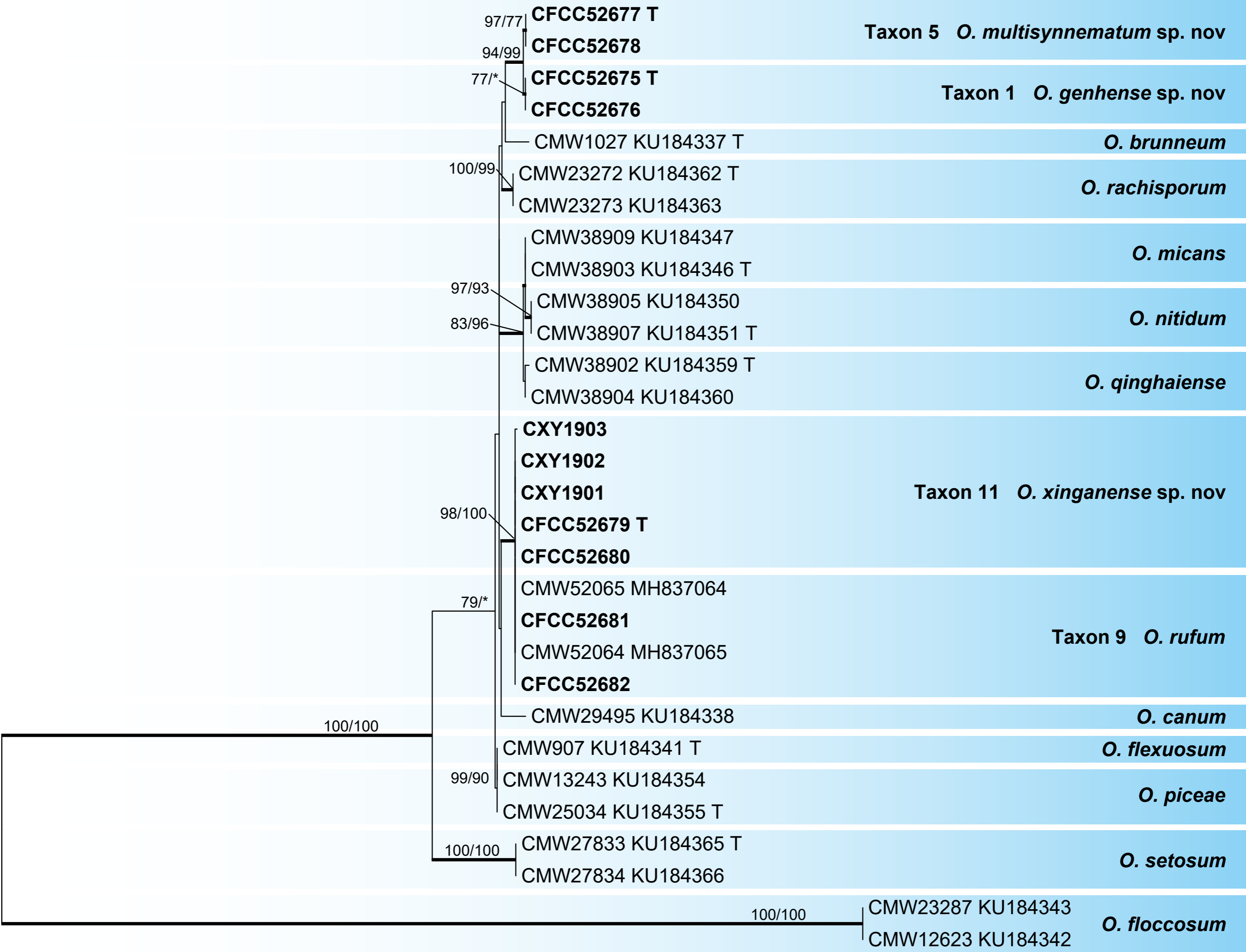

Supplement: Supplementary file 4 — Additional file 4: Figure S3. ML tree of O. piceae complex generated from the CAL sequence data. Sequences generated from this study are printed in bold. Bold branches indicate posterior probability values ≥0.9. Bootstrap values of ML/MP ≥ 70% are recorded at the nodes. T = ex-type isolates [file 43008_2019_25_MOESM4_ESM.pdf]

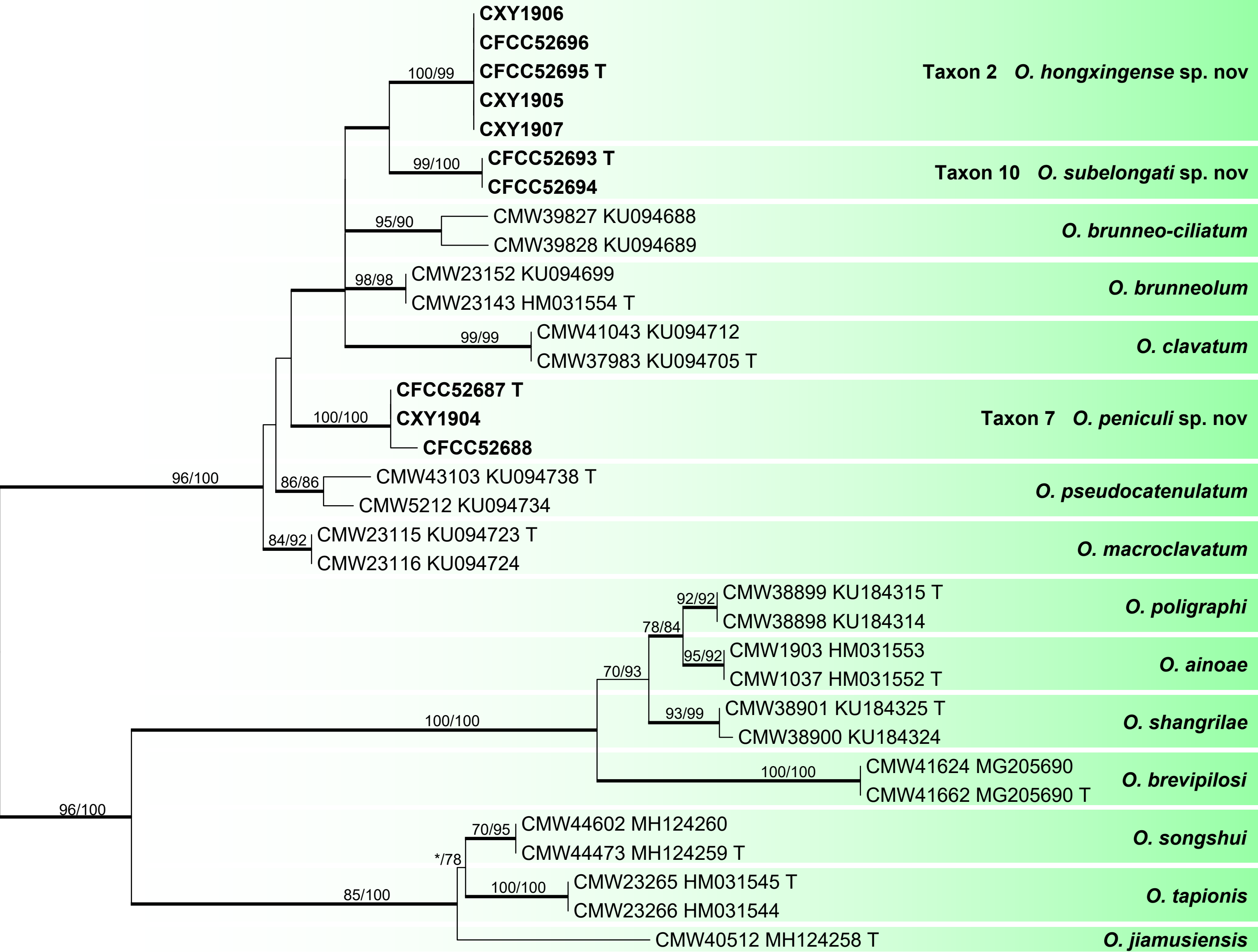

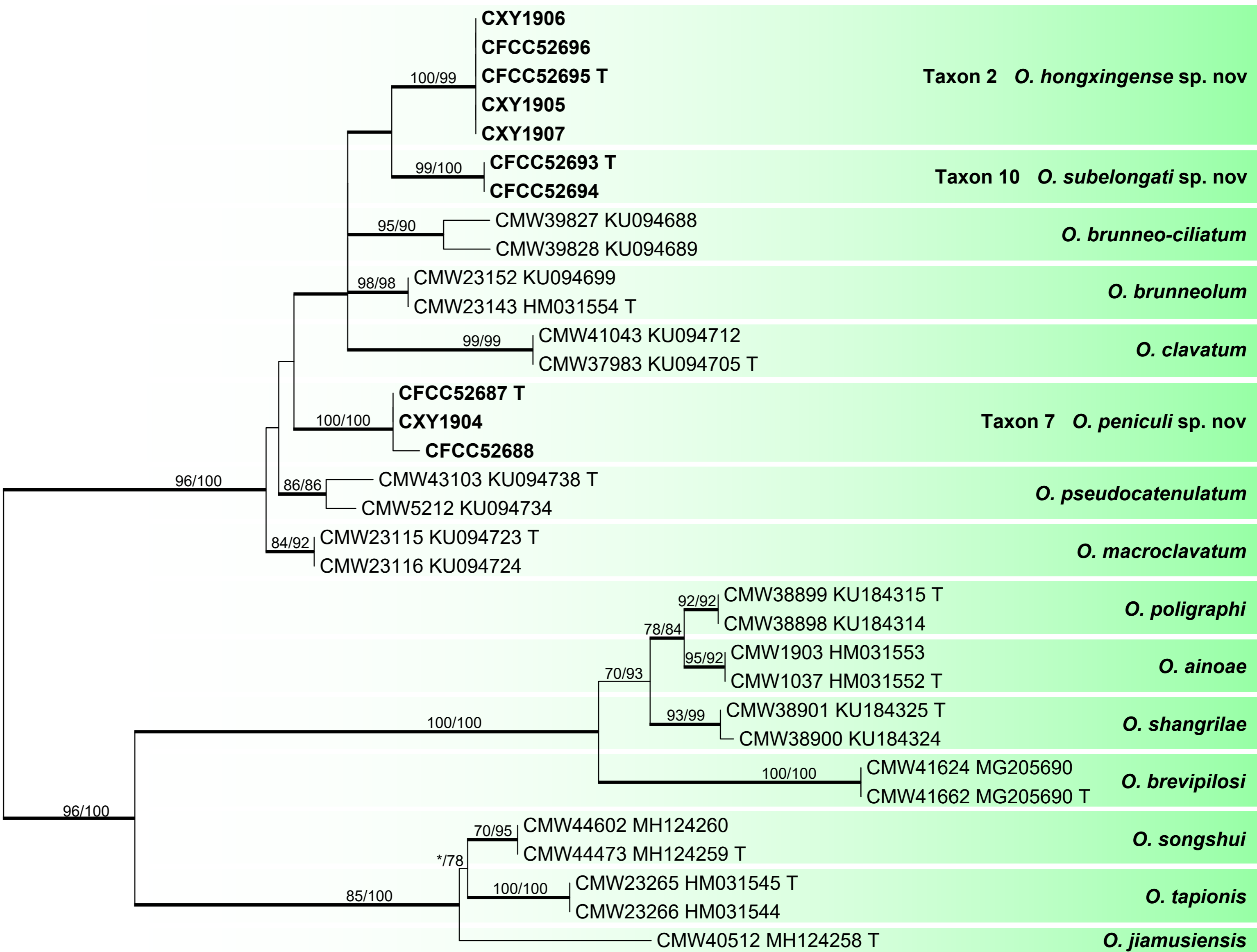

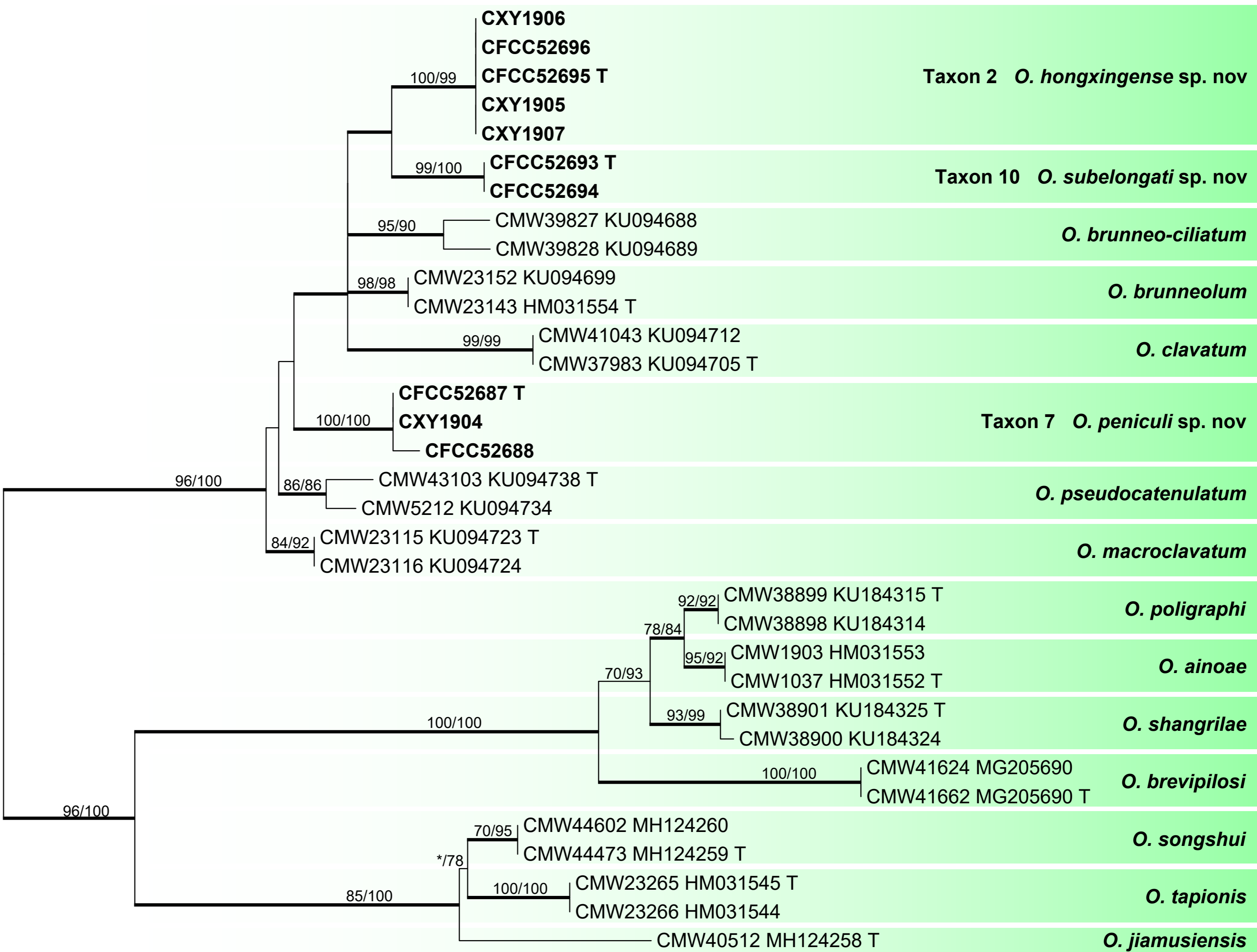

Supplement: Supplementary file 5 — Additional file 5: Figure S4. ML tree of O. clavatum complex generated from the βT sequence data. Sequences generated from this study are printed in bold. Bold branches indicate posterior probability values ≥0.9. Bootstrap values of ML/MP ≥ 70% are recorded at the nodes. T = ex-type isolates [file 43008_2019_25_MOESM5_ESM.pdf]

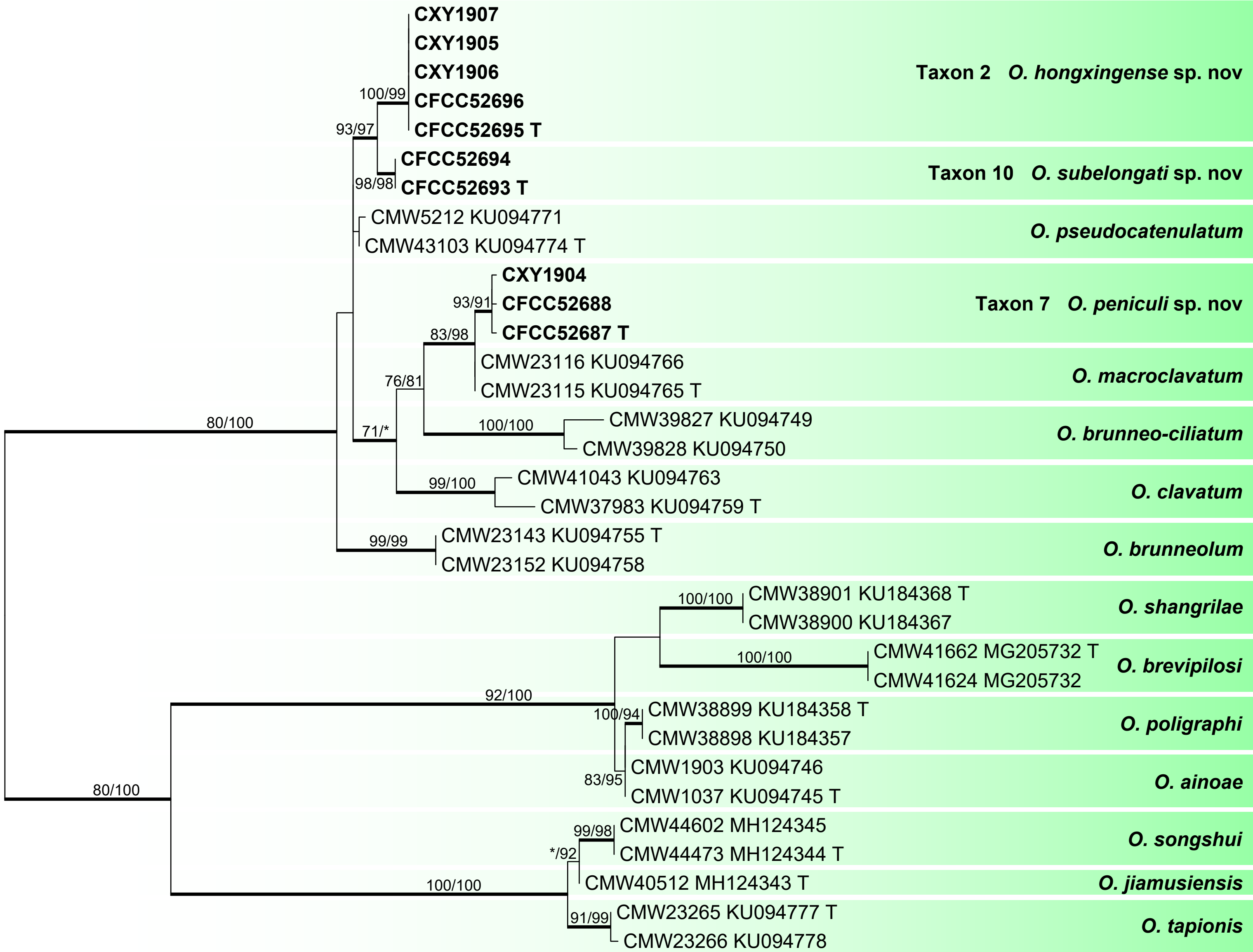

Supplement: Supplementary file 6 — Additional file 6: Figure S5. ML tree of O. clavatum complex generated from the EF-1α sequence data. Sequences generated from this study are printed in bold. Bold branches indicate posterior probability values ≥0.9. Bootstrap values of ML/MP ≥ 70% are recorded at the nodes. T = ex-type isolates [file 43008_2019_25_MOESM6_ESM.pdf]

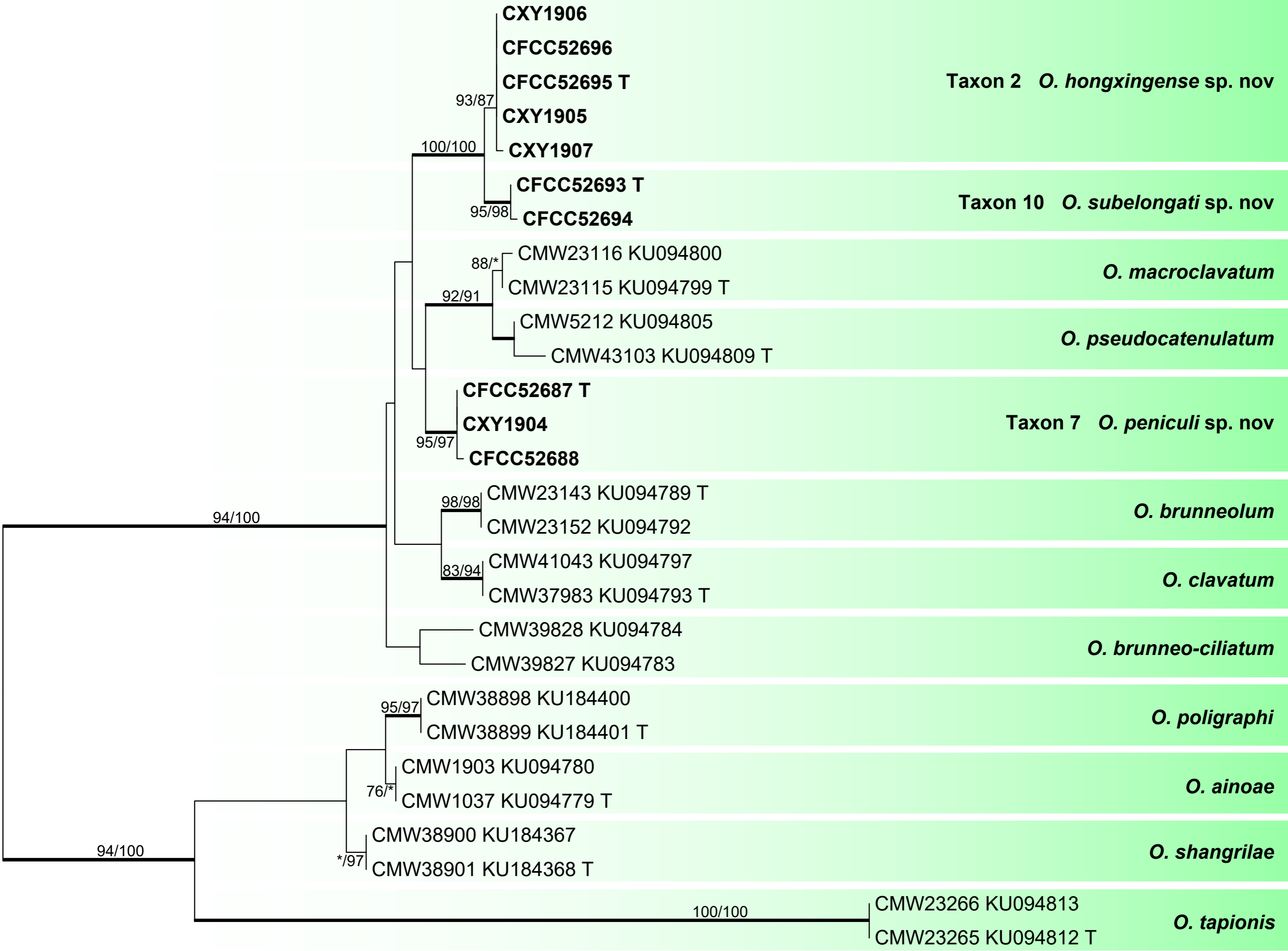

Supplement: Supplementary file 7 — Additional file 7: Figure S6. ML tree of O. clavatum complex generated from the CAL sequence data. Sequences generated from this study are printed in bold. Bold branches indicate posterior probability values ≥0.9. Bootstrap values of ML/MP ≥ 70% are recorded at the nodes. T = ex-type isolates [file 43008_2019_25_MOESM7_ESM.pdf]

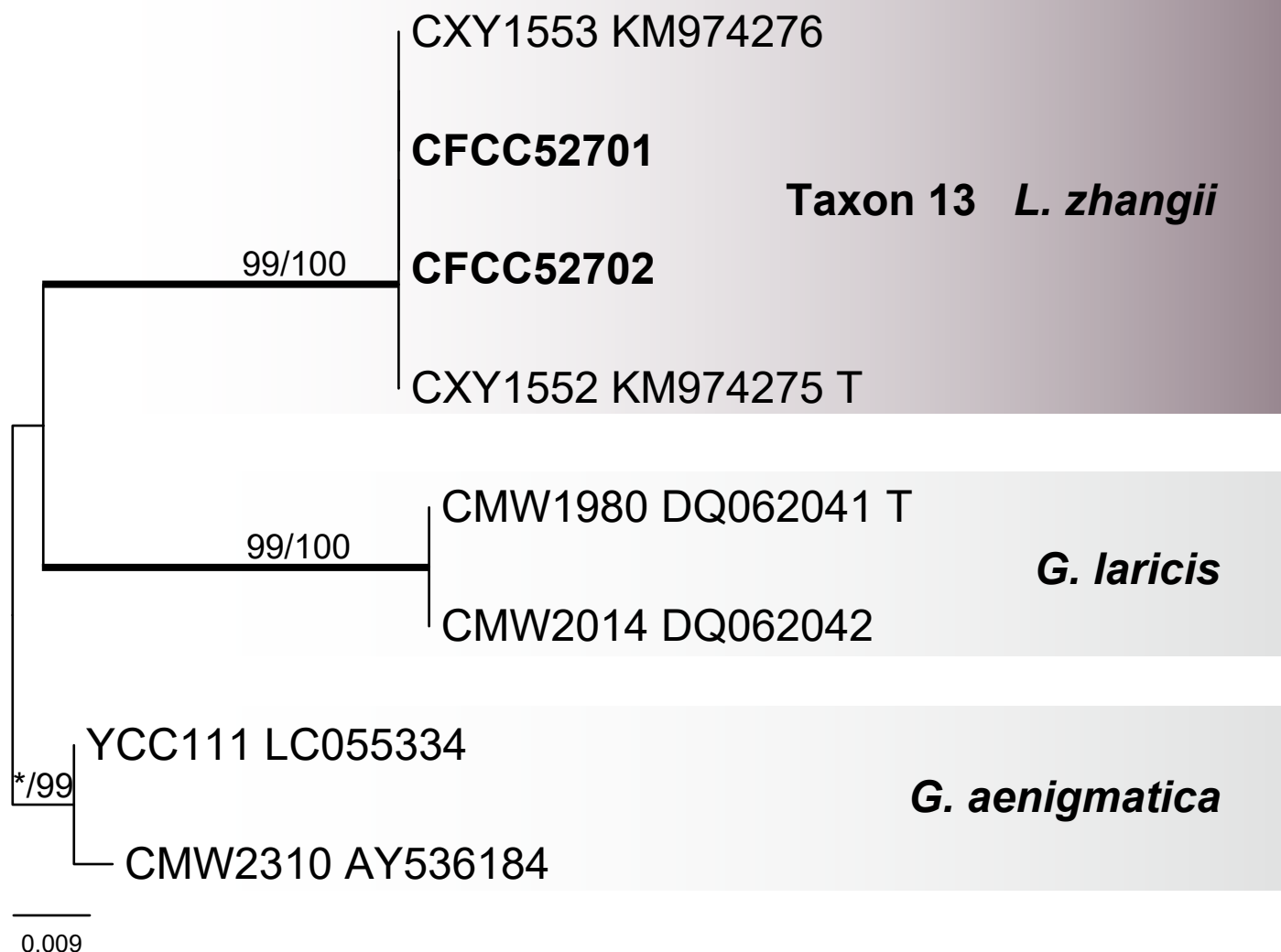

Supplement: Supplementary file 8 — Additional file 8: Figure S7. ML tree of L. zhangii generated from the EF-1α sequence data. Sequences generated from this study are printed in bold. Bold branches indicate posterior probability values ≥0.9. Bootstrap values of ML/MP ≥ 70% are recorded at the nodes. T = ex-type isolates [file 43008_2019_25_MOESM8_ESM.pdf]

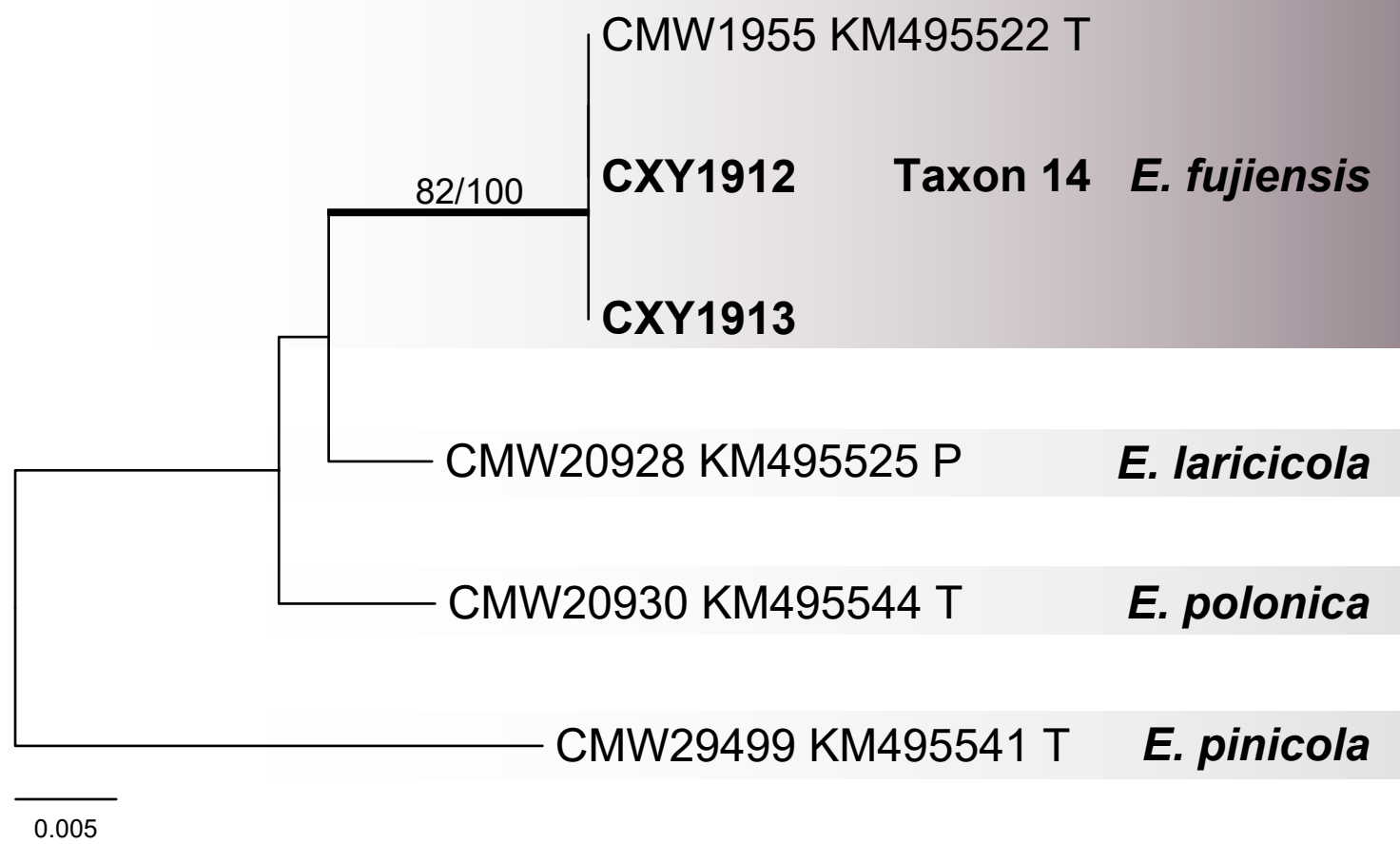

Supplement: Supplementary file 9 — Additional file 9: Figure S8. ML tree of Endoconidiophora generated from the 60S sequence data. Sequences generated from this study are printed in bold. Bold branches indicate posterior probability values ≥0.9. Bootstrap values of ML/MP ≥ 70% are recorded at the nodes. T = ex-type isolates [file 43008_2019_25_MOESM9_ESM.pdf]

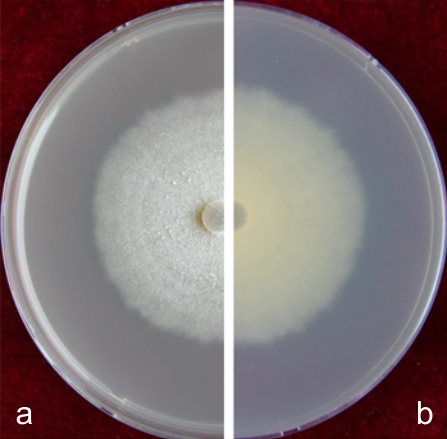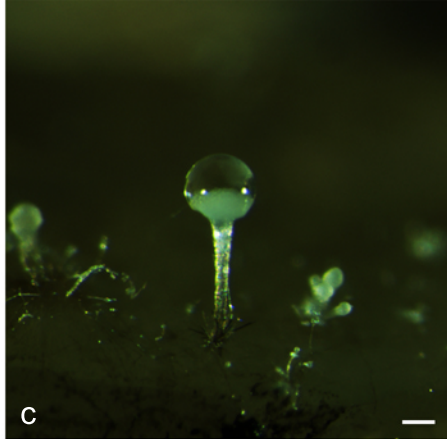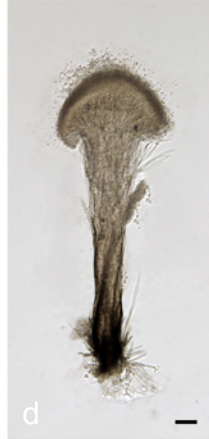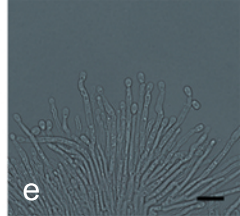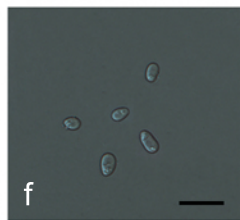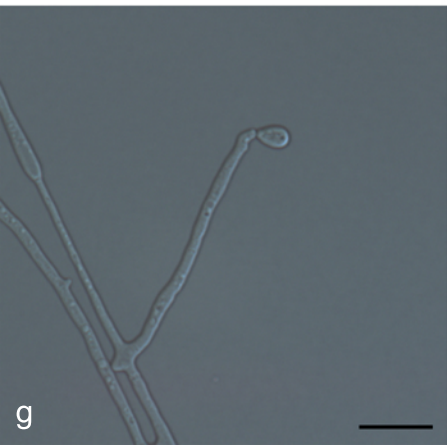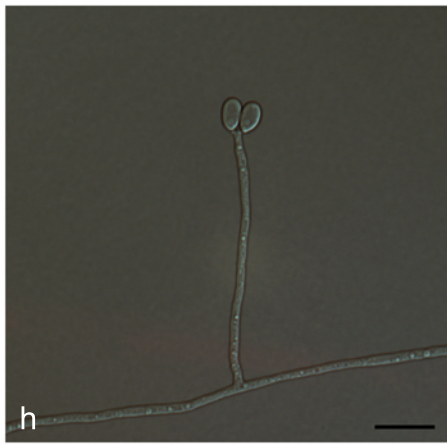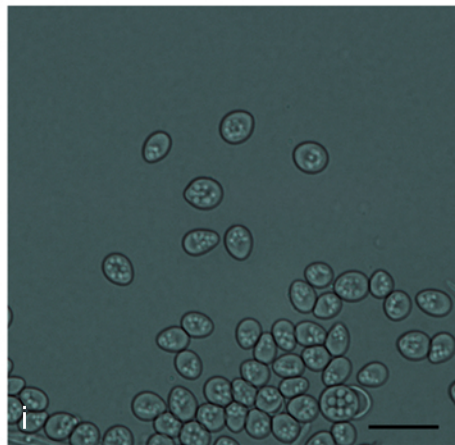

Supplement: Supplementary file 10 — Additional file 10: Figure S9. Morphological characteristics of Ophiostoma rufum (CFCC 52681 Taxon 9). a–b. Ten-day-old cultures on 2% MEA; c–d. Pesotum-like asexual morph; e–f. Conidiogenous cells of pesotum-like asexual morph and conidia; g–i. Hyalorhinocladiella-like asexual morph: conidiogenous cells and conidia. Scale bars: c = 50 μm; d = 20 μm; e–i = 10 μm [file 43008_2019_25_MOESM10_ESM.pdf]
